# A Plug‐and‐Play Platform for Customizing Multivalent Degraders and Degrader‐Drug Conjugates

**DOI:** 10.1002/advs.75658

**Published:** 2026-05-15

**Authors:** Mengqing Zhao, Yan Deng, Jianjian Han, Yong Xie, Kai Bao, Lixin Ma, Lilong Liu, Wuxiang Mao

**Affiliations:** ^1^ State Key Laboratory of Biocatalysis and Enzyme Engineering Hubei Province Key Laboratory of Industrial Biotechnology School of Life Sciences Hubei University Wuhan China; ^2^ Department of Clinical Laboratory Union Hospital Tongji Medical College Huazhong University of Science and Technology Wuhan China; ^3^ Department of Urology Tongji Hospital Tongji Medical College Huazhong University of Science and Technology Wuhan China

**Keywords:** LYTAC, degrader‐drug conjugates, multivalency, protein pairs, plug‐and‐play

## Abstract

Traditional targeted protein degradation (TPD) strategies are largely ineffective against membrane proteins, which constitute over 60% of drug targets. To address this, we develop a modular “plug‐and‐play” UPTAB (Ultrahigh‐affinity Protein pairs fused to Targeting Binders) platform for TPD. The platform leverages orthogonal ultrahigh‐affinity Im/CL protein pairs to assemble complexes between lysosomal trafficking receptor (LTR)‐binding modules and protein of interest (POI)‐binding modules. Three UPTAB configurations were engineered: Type‐I (mono‐targeted), Type‐II (dual‐targeted), and Type‐III (tri‐targeted). In vitro, Type‐I UPTAB achieved near‐complete degradation of EGFR and PD‐L1 across multiple cancer cell lines, with optimal linker length critical for maximal activity. Type‐II and Type‐III UPTAB enabled simultaneous degradation of EGFR/c‐MET, EGFR/PD‐L1, and EGFR/c‐MET/HER2. In a breast cancer xenograft model, Type‐I UPTAB demonstrated approximately 80% tumor growth inhibition, reduced EGFR levels in tumors, and significantly extended survival. Furthermore, we developed degrader‐drug conjugates (DDCs) by site‐specific conjugation of the cytotoxic payload MMAE to UPTAB modules, which retained degradation capacity while exhibiting substantially enhanced anti‐proliferative activity across diverse cancer cell lines. The UPTAB platform combines modular multivalent design, high degradation efficiency, and excellent bioconjugation capability, offering a versatile tool for membrane protein‐targeted degradation and a potential strategy for developing next‐generation cancer therapeutics.

## Graphical abstract

Membrane proteins remain challenging targets for conventional TPD approaches. Here, the authors develop UPTAB, a modular platform leveraging ultrahigh‐affinity orthogonal Im/CL protein pairs for lysosomal degradation of membrane proteins. Mono‐targeted (Type‐I), dual‐targeted (Type‐II), and tri‐targeted (Type‐III) UPTABs enable simultaneous degradation of multiple therapeutic targets. Furthermore, conjugation with MMAE yields DDCs with enhanced antitumor potency, offering a versatile strategy for cancer therapy.

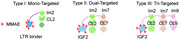

## 1 Introduction

Targeted degradation of pathogenic proteins represents a paradigm shift in therapeutic development, moving beyond simple inhibition toward the complete removal of disease‐causing agents [1, 2]. The remarkable clinical success of proteolysis‐targeting chimeras (PROTACs) has validated this approach for oncology and neurological disorders, with ARV‐471 getting approved by the FDA [3, 4, 5]. These heterobifunctional molecules tether a POI to an E3 ligase, facilitating the POI's ubiquitination and subsequent degradation by the proteasome [6, 7, 8]. A key advancement in PROTAC development has been the implementation of combinatorial chemistry strategies [9, 10, 11]. By pre‐functionalizing E3 ligase ligands and POI ligands with orthogonal reactive groups, researchers can rapidly assemble diverse PROTAC libraries, accelerating PROTAC drug discovery. However, the vast landscape of extracellular and cell membrane proteins, which comprise over 60% of established drug targets, has remained largely inaccessible to these PROTACs [12]. The primary limitation for PROTACs lies in their dependence on intracellular E3 ligases, rendering them ineffective against extracellular proteins or membrane proteins lacking accessible cytosolic domains [13].

Emerging technologies such as lysosome‐targeting chimeras (LYTACs), antibody‐based PROTACs (AbTACs), and autophagy‐inducing antibodies (AUTABs) for membrane protein degradation have expanded the reach of TPD beyond intracellular targets [14, 15, 16, 17, 18, 19]. These strategies depend on the essential functions of cell‐surface receptors; however, the mechanisms governing receptor endocytosis vary among different receptors. For instance, ligand binding to the insulin‐like growth factor 2 receptor (IGF2R) triggers a conformational change that promotes internalization. In contrast, sortilin and the transferrin receptor (TfR) constitutively traffic between the cell surface and the endosome‐lysosome [20, 21, 22]. Notably, the degradation efficacy achieved in these TPD strategies varies significantly depending on the LTR. These approaches leverage alternative cellular machinery to deplete previously “undruggable” extracellular and membrane proteins [23, 24]. A key advance came in 2025, when Zhou et al. revealed that TransTACs containing a cathepsin‐sensitive linker reduced POI recycling and improved degradation, establishing lysosomal trafficking as a central strategy for enhancing membrane protein degradation [25].

Membrane proteins frequently act in concert to drive tumor proliferation and survival while also contributing to therapeutic resistance, highlighting the clinical potential of multi‐target degradation approaches. To address the challenge of simultaneous dual‐targeted degradation, Wei et al. developed FolTACs‐dual, a rationally designed modular system that enables the degradation of two membrane proteins simultaneously, such as EGFR/HER2 and PD‐L1/VISTA [26]. Despite these advances, existing technologies are typically limited to mono‐ or dual‐targeted applications and lack a multivalent platform that can be rapidly adapted for multi‐targeted degradation. Protein‐based self‐assembly has emerged as a versatile and modular platform for engineering biologic agents, with applications in biosensors, biocatalysis, drug delivery, and vaccine development [27]. This modularity has been advanced by the development of bio‐orthogonal conjugation strategies, such as the SpyCatcher‐SpyTag system, which facilitates the site‐specific assembly of fused proteins onto therapeutic scaffolds [28]. Our previous research systematically investigated the orthogonality and ultrahigh avidity between cognate immunity proteins (Im) and engineered DNase domains of CE variants (CL), and successfully demonstrated the self‐assembly application of Im/CL systems [29]. To this end, we sought to develop a “plug‐and‐play” platform for customizing multivalent degraders based on UPTABs.

Given that antibody‐drug conjugates (ADCs) employing cleavable linkers depend on efficient lysosomal trafficking, we sought to leverage the lysosomal delivery capacity of UPTABs to enhance payload delivery. Thus, we developed DDCs, a novel class of bifunctional therapeutics that hybridize UPTABs and ADC platforms to achieve more efficient cytosolic delivery of drug payloads [30]. In this work, UPTAB leverages the engineered Im/CL system to create a highly efficient and multivalent degradation platform. We demonstrate that Im‐ or CL‐fusions enhance recombinant protein yields in *E. coli* by up to 8‐fold, and that UPTAB exhibits superior degradation activity compared to a known direct fusion by up to 5‐fold, achieving over 95% target degradation. Furthermore, conjugation of cytotoxic payloads to degraders yielded DDCs, which substantially enhanced degrader potency across a panel of five cancer cell lines. Collectively, our findings establish DDCs as a potential therapeutic modality that may offer a new strategy to both improve payload delivery and expand the therapeutic applications of membrane protein degraders.

## 2 Results and Discussion

### 2.1 Design of UPTAB Platforms for Plug‐and‐Play Customization of Multivalent Degraders and Degrader‐Drug Conjugates

**FIGURE 1:**
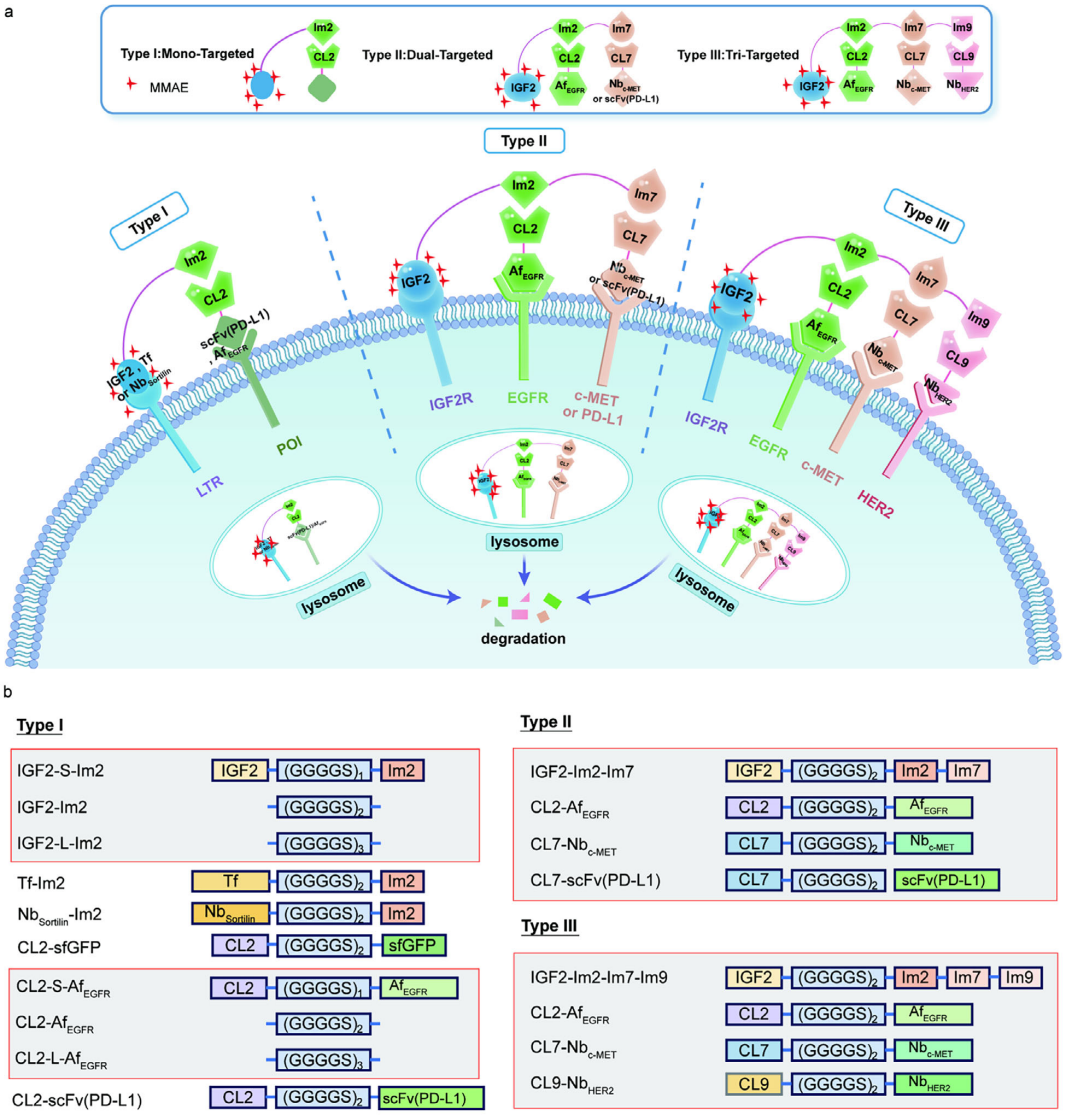
Design principles of UPTAB platforms for plug‐and‐play customization of multivalent degraders and degrader‐drug conjugates. (a) Schematic illustration of Im‐fused LTR binders and CL‐fused POI binders employed for membrane protein degradation via the lysosomal pathway. LTR‐binding scaffolds include mono‐targeted (IGF2‐Im2, Tf‐Im2, Nb_Sortilin_‐Im2), dual‐targeted (IGF2‐Im2‐Im7), and tri‐targeted (IGF2‐Im2‐Im7‐Im9) configurations. Corresponding POI‐binding fusions comprise CL2‐Af_EGFR_ (EGFR‐targeting), CL2‐scFv(PD‐L1) (PD‐L1‐targeting), CL7‐Nb*_c_*_‐MET_ (c‐MET‐targeting), and CL9‐Nb_HER2_ (HER2‐targeting). Assembly of these orthogonal Im/CL pairs enables modular, multivalent targeting of membrane proteins for lysosomal degradation. DDCs were generated by conjugation with the cytotoxic payload MMAE to enhance the killing of cancer cells. (b) Schematic representation of Im‐fused LTR binders, CL2‐fused GFP, and CL‐fused POI binders.

The UPTAB platform comprises two modular components: “degrader” modules, consisting of LTR‐binding domains fused to Im proteins, and “targeted” modules, comprising POI‐binding domains fused to CL proteins. This modular architecture enables flexible and versatile customization of degraders for diverse targets. Given that IGF2R, TfR, and Sortilin are highly expressed in cancer cells, we selected these LTRs for membrane protein degradation [20, 31, 32, 33]. To demonstrate the design principles of UPTAB, we hijacked three LTRs to degrade therapeutically relevant membrane proteins, including epidermal growth factor receptor (EGFR), programmed death‐ligand 1 (PD‐L1), cellular‐mesenchymal epithelial transition factor (c‐MET), and human epidermal growth factor receptor 2 (HER2) (Figure 1) [34, 35, 36, 37, 38, 39, 40, 41].

The core technology leverages ultrahigh‐affinity orthogonal protein pairs (Im2/CL2, Im7/CL7, and Im9/CL9) to assemble complexes between LTR‐binding and POI‐binding domains (Figure 1a). In the Type‐I UPTAB configuration, orthogonal LTR‐binding domains were fused to the Im2 domain: IGF2 (targeting IGF2R), transferrin (Tf, targeting TfR), and Nb_Sortilin_ (targeting Sortilin), yielding IGF2‐Im2, Tf‐Im2, and Nb_Sortilin_‐Im2, respectively. Separately, POI‐binding domains were fused to the CL2 domain, generating CL2‐Af_EGFR_ and CL2‐scFv(PD‐L1). In this configuration, the LTR‐Im2 fusion recruits a CL2‐POI binder, tethering the POI to LTR to facilitate its internalization and degradation. The Type‐I UPTAB is mono‐targeted, mediating degradation of a single target per complex. To achieve dual‐targeted degradation, we developed a Type‐II UPTAB. In this design, IGF2 was fused to both Im2 and Im7, while the POI‐binding domains Af_EGFR_ and Nb_c‐MET_ or scFv(PD‐L1) were fused to CL2 and CL7, respectively. Leveraging the orthogonality of the Im2/CL2 and Im7/CL7 pairs, a protein complex assembles that simultaneously engages EGFR/c‐MET or EGFR/PD‐L1, mediating their concurrent degradation.

For tri‐targeted degradation, we engineered Type‐III UPTAB by extending the scaffold to IGF2‐Im2‐Im7‐Im9, incorporating three orthogonal protein‐binding sites. The corresponding POI‐binding domains (Af_EGFR_, Nb*_c_*_​‐MET_, and Nb_HER2_) were fused to CL2, CL7, and CL9, respectively. This design enables assembly of a multivalent protein complex that induces simultaneous degradation of EGFR, c‐MET, and HER2. Furthermore, by conjugating valine‐citrulline monomethyl auristatin E (vcMMAE) to IGF2‐Im2, IGF2‐Im2‐Im7, or IGF2‐Im2‐Im7‐Im9 scaffolds, we demonstrated that the UPTAB platform enables efficient generation of modular DDCs. These conjugates exhibited potent antitumor efficacy through endocytosis‐mediated payload release. Schematic representations of Im‐fused LTR binders, CL2‐GFP, and CL‐fused POI binders are shown in Figure 1b. To optimize flexibility and interdomain spacing, we varied linker length by incorporating one to three GGGGS repeats between IGF2 and Im2, as well as between CL2 and Af_EGFR_.

### 2.2 Synthesis and Optimization of the Type‐I UPTAB Platform for Mono‐Targeted Internalization and Degradation

**FIGURE 2:**
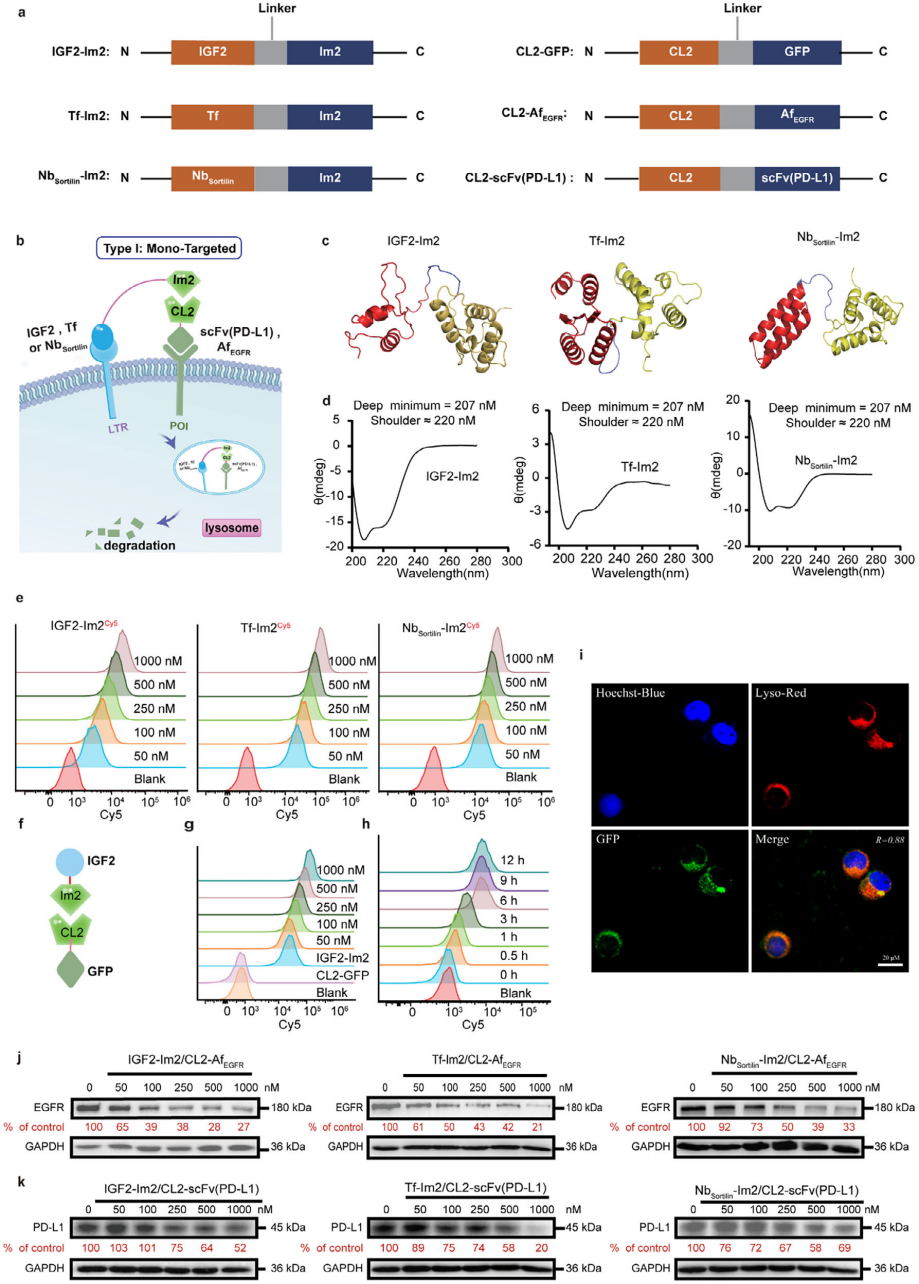
Characterizations and initial evaluation of Im2‐fused LTR binders and the Type‐I UPTAB platform. (a) Schematic representation of gene constructs used in this study, including Im2‐fused LTR binders (IGF2‐Im2, Tf‐Im2, Nb_Sortilin_‐Im2) and CL2‐fused constructs (CL2‐GFP, CL2‐Af_EGFR_, CL2‐scFv(PD‐L1)). (b) Schematic illustration of EGFR or PD‐L1 degradation mediated by Type‐I UPTAB. (c) AlphaFold3‐predicted structures of Im2‐fused LTR binders. LTR‐binding domains (IGF2, Tf, Nb_Sortilin_) are shown in red, Im2 in yellow, and GS linkers in blue. (d) CD spectra of Im2‐fused LTR binders in 10 mM potassium phosphate buffer (pH 7.2). (e) Flow cytometric analysis of cellular uptake of fluorescently labeled LTR binders (IGF2‐Im2^Cy5^, Tf‐Im2^Cy5^, Nb_Sortilin_‐Im2^Cy5^) in HeLa cells after 6 h incubation at indicated concentrations (0–1000 nM). (f) Schematic illustration of complex assembly between IGF2‐Im2 and CL2‐GFP. (g) Cellular uptake of IGF2‐Im2^Cy5^ (0–1000 nM) following pre‐assembly with CL2‐GFP (0–1000 nM) for 6 h in HeLa cells. Unpaired IGF2‐Im2^Cy5^ (50 nM) and CL2‐GFP (50 nM) alone served as negative controls. (h) Time‐dependent cellular uptake of pre‐assembled IGF2‐Im2^Cy5^ with CL2‐GFP (100 nM each) over 0.5–12 h in HeLa cells, analyzed by flow cytometry. (i) Representative CLSM images of HeLa cells treated with pre‐assembled IGF2‐Im2/CL2‐GFP (500 nM each) for 24 h. GFP (green), LysoTracker (red), and Hoechst (blue). Scale bar = 20 µm. (j) WB analysis of EGFR degradation in HeLa cells treated with indicated combinations of Im2‐fused LTR binders and CL2‐Af_EGFR_ (0–1000 nM) for 24 h (n = 3 independent experiments). (k) WB analysis of PD‐L1 degradation in MDA‐MB‐231 cells treated with indicated combinations of Im2‐fused LTR binders and CL2‐scFv(PD‐L1) (0–1000 nM) for 48 h (n = 3 independent experiments).

We initially hypothesized that the Type‐I UPTAB could function as a “plug‐and‐play” platform for customizing degraders capable of mediating broad and efficient lysosomal delivery and subsequent degradation of membrane proteins (Figure 2b). To engineer this modular system, we fused Im2 and CL2 domains to LTR‐binding domains and POI‐binding domains, respectively. This strategy yielded six gene constructs, including Im2‐fused LTR binders (IGF2‐Im2, Tf‐Im2, Nb_Sortilin_‐Im2) and CL2‐fused constructs (CL2‐GFP, CL2‐Af_EGFR_, CL2‐scFv(PD‐L1)) (Figure 2a). To address the low expression levels of LTR‐binding domains in *E. coli*, each construct was equipped with an N‐terminal SUMO tag to enhance soluble protein production [42]. Following purification by Ni‐NTA affinity chromatography, the SUMO tags were cleaved, and the identity and purity of the final proteins were confirmed by SDS‐PAGE (Figure S1). The structural integrity of purified fusions was verified using circular dichroism (CD) spectroscopy (Figure 2d). The spectra indicated that all three proteins adopted predominantly α‐helical secondary structures, consistent with their predicted models generated by AlphaFold3 (Figure 2c).

To directly assess the cellular uptake potential of these LTR‐binder fusions, we fluorescently labeled IGF2‐Im2, Tf‐Im2, and Nb_Sortilin_‐Im2 with Cy5. Flow cytometry analysis revealed that all three constructs were internalized in a dose‐dependent manner (Figure 2e and Figure S2), confirming that fusion of Im2 to LTR‐binding domains does not impair their endocytic capacity. However, whether this delivery pathway could facilitate subsequent trafficking to the lysosome remained unknown. To investigate this, we generated CL2‐GFP for live‐cell visualization of cargo delivery. The structural integrity of purified CL2‐GFP was verified by CD spectroscopy (Figure S3b), which revealed a mixed α‐helical/β‐sheet structure consistent with its predicted model (Figure S3a).

We next examined the uptake of IGF2‐Im2/CL2‐GFP complexes in live mammalian cells using flow cytometry and confocal laser scanning microscopy (CLSM). Following assembly with CL2‐GFP (Figure 2f), IGF2‐Im2 mediated complex internalization in both concentration‐ and time‐dependent manners (Figure 2g,h, and Figure S4). Flow cytometric analysis revealed that CL2‐GFP binding did not impair IGF2‐Im2 internalization, with maximal endocytosis of IGF2^Cy5^ or CL2‐GFP achieved after 6 h incubation (Figure 2h and Figure S4b). Efficient internalization of IGF2‐Im2/CL2‐GFP in HeLa cells was observed, with approximately 10‐fold enhancement in fluorescence signal (Figure 2g). Subsequently, live‐cell CLSM was performed to track the intracellular localization of assembled IGF2‐Im2/CL2‐GFP complexes. Cells co‐treated with IGF2‐Im2 and CL2‐GFP exhibited clear co‐localization of the green fluorescent signal with LysoTracker Red, a lysosomal marker (Figure 2i), demonstrating that CL2‐GFP cargo was successfully trafficked to lysosomes. Together, the synergistic combination of efficient uptake, high‐affinity binding, and successful lysosomal delivery supported the feasibility of using Type‐I UPTABs for lysosomal degradation of membrane proteins. To establish Type‐I UPTAB as a general and modular platform for membrane protein degradation, we constructed CL2‐Af_EGFR_ and CL2‐scFv(PD‐L1). Successful purification of these fusion proteins was confirmed by SDS‐PAGE (Figure S1a), and their secondary structures were characterized by CD spectroscopy (Figure S5b), which revealed a predominantly α‐helical structure for CL2‐Af_EGFR_ and a mixed α‐helical/β‐sheet structure for CL2‐scFv(PD‐L1). These findings were consistent with structural predictions from AlphaFold3 (Figure S5a).

We next investigated whether Type‐I UPTAB could mediate degradation of EGFR, a receptor tyrosine kinase overexpressed in many tumors whose degradation could offer a therapeutic strategy [43, 44]. Western blot (WB) analysis revealed that HeLa cells treated with assembled Im2‐fused LTR‐binding domains (IGF2‐Im2, Tf‐Im2, or Nb_Sortilin_‐Im2) with CL2‐Af_EGFR_ exhibited significant, dose‐dependent EGFR degradation (Figure 2j). The IGF2‐Im2/CL2‐Af_EGFR_ complex was the most potent, achieving near‐complete degradation at 100–500 nM. This superior efficacy correlates with the higher affinity of IGF2 for IGF2R (Kd = 4.6 nM), compared to the affinities of Tf for TfR and Nb_Sortilin_ for Sortilin (Kd ≈ 20 nM each) [20, 34]. Consequently, IGF2‐Im2 was selected as the primary “degrader” module for subsequent studies. Given the important roles of PD‐L1 in cancer immunotherapy, we extended this platform to degrade PD‐L1 using CL2‐scFv(PD‐L1) as “targeted” modules, thereby expanding the target scope (Figure 2k) [45]. Dose‐dependent PD‐L1 degradation was similarly observed, with maximum degradation efficiencies of 48% (IGF2R‐mediated), 80% (TfR‐mediated), and 42% (Sortilin‐mediated). Consistent with previous reports, the overall degradation efficacy is governed by a “composite parameter” proportional to (LTR surface expression) x (binding affinity) x (target expression) [31]. For IGF2R, high affinity but lower expression in MDA‐MB‐231; for TfR, lower affinity but higher expression in MDA‐MB‐231. These two LTRs both yield similar target internalization and degradation. Sortilin, with both low affinity and low expression, shows the weakest activity (Figure S6).

### 2.3 Type‐I UPTAB Exhibits Enhanced Expression and Superior Degradation Activity Compared to Direct Fusion Constructs

**FIGURE 3:**
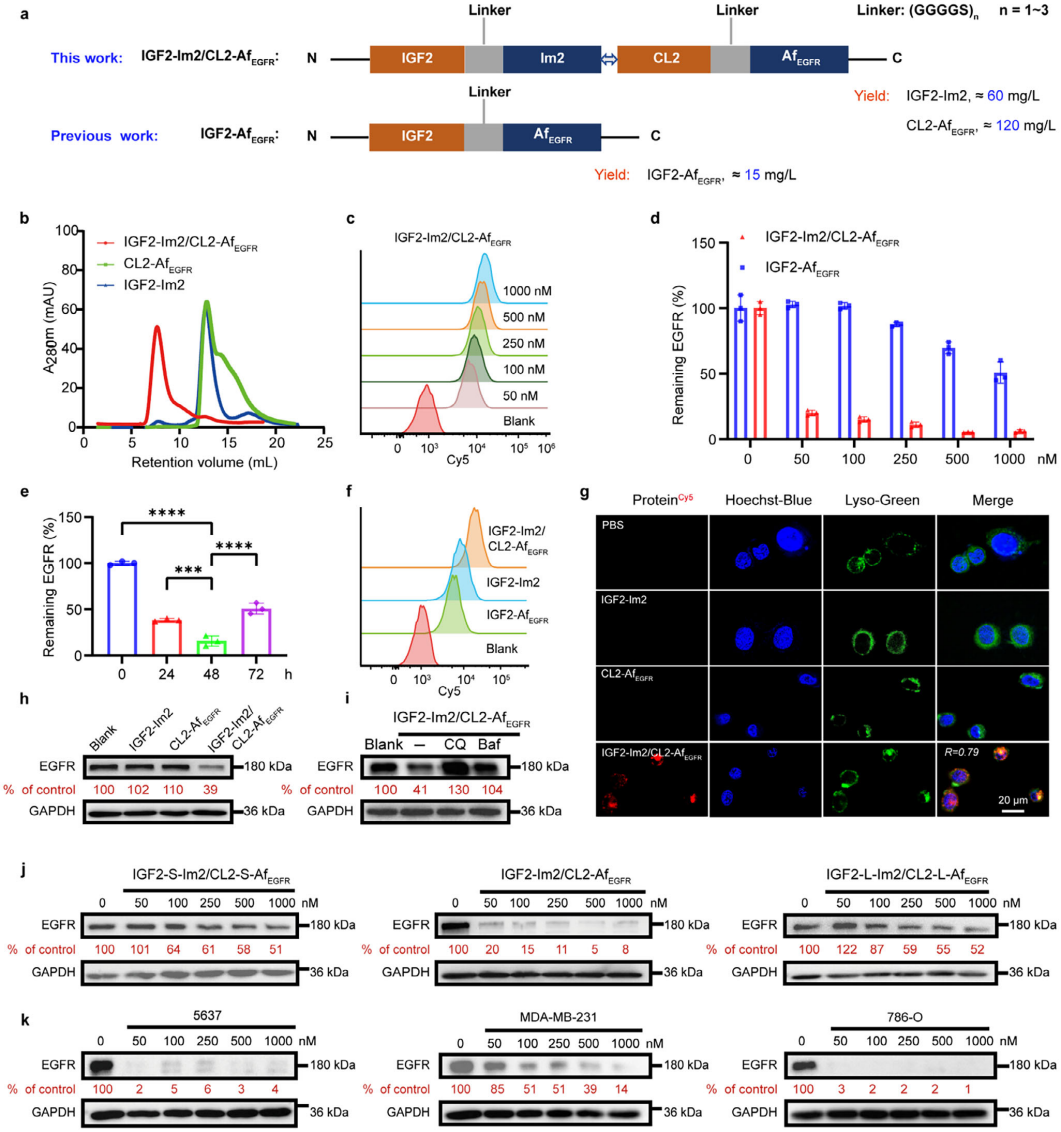
Internalization and lysosomal degradation of EGFR by IGF2‐Im2/CL2‐Af_EGFR_. (a) Comparison of recombinant protein expression yields in *E. coli* for IGF2‐Im2, CL2‐Af_EGFR_, and the direct fusion construct IGF2‐Af_EGFR_ (n = 3 independent experiments). (b) SEC analysis of complex formation following incubation of IGF2‐Im2 (blue) and CL2‐Af_EGFR_ (green). Absorbance monitored at 280 nm. (c) Flow cytometric analysis of cellular uptake of fluorescently labeled IGF2‐Im2^Cy5^/CL2‐Af_EGFR_ in HeLa cells after 6 h incubation at indicated concentrations (0–1000 nM). (d) Quantification of EGFR levels in HeLa cells treated with assembled IGF2‐Im2/CL2‐Af_EGFR_ or IGF2‐Af_EGFR_ at indicated concentrations (0–1000 nM) for 48 h (n = 3 independent experiments). PBS‐treated groups served as normalization controls. (e) Time‐dependent EGFR degradation in HeLa cells treated with IGF2‐Im2/CL2‐Af_EGFR_ (100 nM) for 0–72 h (n = 3 independent experiments). (f) Cellular uptake of IGF2‐Im2^Cy5^ (100 nM) following pre‐assembly with CL2‐Af_EGFR_^Cy5^ (100 nM) for 6 h in HeLa cells. Unpaired IGF2‐Im2^Cy5^ (100 nM) and IGF2‐Af_EGFR_^Cy5^ (100 nM) served as controls. (g) CLSM images showing cellular uptake and lysosomal localization of IGF2‐Im2/CL2‐Af_EGFR_ in HeLa cells. Cells were treated with IGF2‐Im2 alone, Cy5‐labeled CL2‐Af_EGFR_ alone, or assembled IGF2‐Im2/CL2‐Af_EGFR_^Cy5^ for 24 h. Cy5‐labeled protein (red), LysoTracker (green), Hoechst (blue). Scale bar = 20 µm. (h) WB analysis of EGFR levels in HeLa cells treated with IGF2‐Im2 alone, CL2‐Af_EGFR_ alone, or assembled IGF2‐Im2/CL2‐Af_EGFR_ (100 nM) for 48 h (n = 3 independent experiments). (i) WB analysis of EGFR levels in HeLa cells treated with IGF2‐Im2/CL2‐Af_EGFR_ (100 nM) for 24 h in the presence or absence of lysosomal inhibitors (50 µM CQ or 50 nM Baf) (n = 3 independent experiments). (j) WB analysis of EGFR levels in HeLa cells treated with Type‐I UPTAB variants containing different linker lengths (100 nM) for 48 h: IGF2‐S‐Im2/CL2‐S‐Af_EGFR_, IGF2‐Im2/CL2‐Af_EGFR_, and IGF2‐L‐Im2/CL2‐L‐Af_EGFR_ (n = 3 independent experiments). “S” denotes short linker ((GGGGS)_1_); “L” denotes long linker ((GGGGS)_3_). (k) Concentration‐dependent EGFR degradation in MDA‐MB‐231, 786‐O, and 5637 cells treated with IGF2‐Im2/CL2‐Af_EGFR_ (1:1 molar ratio) at indicated concentrations (0–1000 nM) for 48 h (n = 3 independent experiments). Data are shown as mean ± SD. Statistical significance was assessed using one‐way ANOVA with Tukey's multiple comparisons test; ^*^ *p* < 0.05; ^**^ *p* < 0.01; ^***^ *p* < 0.001; ^****^ *p* < 0.0001.

Affibodies and nanobodies represent attractive binding domains for TPD applications due to their small size, high stability, and robust expression in *E. coli* [34]. Given that linker length is a critical determinant of degradation efficiency in targeted platforms such as PROTACs, we engineered fusion proteins with shorter (S) or longer (L) linkers [46, 47]. Specifically, we generated IGF2‐S‐Im2 and IGF2‐L‐Im2, containing (GGGGS)​_1_ and (GGGGS)_3_ linkers, respectively, as well as CL2‐S‐Af_EGFR_ and CL2‐L‐Af_EGFR_ with corresponding linker lengths (Figures 1b and 3a). For benchmarking against a conventional bifunctional degrader, we also prepared a known direct fusion construct, IGF2‐Af_EGFR_ [34]. All proteins were expressed in *E. coli* and purified by Ni‐NTA affinity chromatography followed by SUMO tag cleavage. Notably, the modular components of the Type‐I UPTAB platform exhibited substantially higher expression yields compared to the direct fusion: IGF2‐Im2 and CL2‐Af_EGFR_ achieved yields of approximately 60 and 120 mg/L of bacterial culture, respectively, whereas IGF2‐Af_EGFR_ yielded only ∼15 mg/L. All purified proteins were obtained in soluble form and used directly in subsequent experiments without the need for time‐consuming refolding procedures.

To characterize the self‐assembly of IGF2‐Im2 and CL2‐Af_EGFR_ into a functional complex, we performed size‐exclusion chromatography (SEC) analysis. The elution profile of the mixture revealed a distinct peak shift (red) compared to IGF2‐Im2 (blue) or CL2‐Af_EGFR_ (green), confirming specific complex formation under ambient conditions (Figure 3b). This interaction was further validated by Ni‐NTA pull‐down assays, in which untagged IGF2‐Im2 co‐eluted with His‐tagged CL2‐Af_EGFR_ in the presence of 200 mM imidazole, demonstrating stable complex assembly (Figure S7). We next evaluated whether the complex formation affected cellular uptake. Flow cytometric analysis revealed that CL2‐Af_EGFR_ binding did not impair IGF2‐Im2^Cy5^ internalization; rather, the IGF2‐Im2^Cy5^/CL2‐Af_EGFR_ complex was internalized in a concentration‐dependent manner (Figure 3c). Notably, Cy5‐labeled IGF2‐Im2/CL2‐Af_EGFR_ exhibited approximately 20‐fold enhancement in fluorescence signal compared to Cy5‐labeled IGF2‐Im2 or IGF2‐Af_EGFR_ (Figure 3f), indicating that the UPTAB configuration promotes superior cellular uptake. Consistent with enhanced cellular uptake, WB analysis revealed that IGF2‐Im2/CL2‐Af_EGFR_ mediated substantially more potent EGFR degradation than IGF2‐Af_EGFR_. At a low concentration (50 nM), the IGF2‐Im2/CL2‐Af_EGFR_ achieved up to 5‐fold greater EGFR degradation (Figure 3d and Figure S8a), likely attributable to the enhanced flexibility and optimal geometry afforded by modular design. These results established that the Type‐I UPTAB functions not only as a versatile “plug‐and‐play” platform but also as a highly efficient degrader.

To further establish the mechanistic basis and therapeutic potential of this platform, we systematically characterized its degradation capacity. Treatment with 100 nM IGF2‐Im2/CL2‐Af_EGFR_ resulted in approximately 50% EGFR degradation within 24 h, with levels decreasing to 11% of control by 48 h (Figure 3e). As no further sustained degradation was observed at 72 h, the 48 h timepoint was selected as optimal for subsequent experiments (Figure S8b). CLSM demonstrated clear co‐localization of the IGF2‐Im2/CL2‐Af_EGFR_^Cy5^ complex with LysoTracker Green, confirming successful trafficking of EGFR to lysosomes (Figure 3g). Cells treated with IGF2‐Im2 or CL2‐Af_EGFR_^Cy5^ alone exhibited no detectable red signal, underscoring the requirement for ternary complex assembly between the “targeting” and “degrader” modules. To directly confirm that lysosomal trafficking leads to target degradation, we assessed EGFR levels by WB analysis. Cells treated with the assembled IGF2‐Im2/CL2‐Af_EGFR_ complex exhibited significant EGFR degradation, whereas control groups receiving either IGF2‐Im2 or CL2‐Af_EGFR_ showed no degradation (Figure 3h). These results confirm that EGFR internalization and degradation are strictly dependent on the formation of the ternary complex comprising IGF2R, IGF2‐Im2/CL2‐Af_EGFR_, and EGFR. To verify engagement of the lysosomal degradation pathway, cells were pre‐treated with the lysosomal inhibitors bafilomycin A1 (Baf) and chloroquine (CQ). Both interventions significantly attenuated EGFR degradation, confirming that the IGF2‐Im2/CL2‐Af_EGFR_ complex mediates target elimination through lysosomal proteolysis (Figure 3i). Furthermore, to more convincingly demonstrate that the observed degradation depends specifically on the proposed lysosomal trafficking receptor (IGF2R), we performed experiments in HeLa IGF2R knockout (KO) cells (Figure S9a). Compared with degradation observed in HeLa wild‑type (WT) cells, degradation was markedly inhibited in HeLa IGF2R‑KO cells (Figure S9b,c). This indicates that the degradation in this study is mediated by an IGF2R‑dependent mechanism.

We next examined the impact of linker length on degradation efficiency. While all Type‐I UPTAB variants with varying GGGGS repeats retained EGFR‐degrading activity, both the short‐linker (IGF2‐S‐Im2/CL2‐S‐Af_EGFR_, (GGGGS)_1_) and long‐linker (IGF2‐L‐Im2/CL2‐L‐Af_EGFR_, (GGGGS)_3_) configurations were less efficient than the original constructs containing (GGGGS)_2_ linkers (Figure 3j). This highlights the importance of optimal spacing for maximal degradation activity. Finally, to evaluate the broader applicability of the Type‐I UPTAB platform, we assessed its efficacy across multiple cancer cell lines, including breast cancer MDA‐MB‐231, renal cell carcinoma 786‐O, and bladder cancer 5637 cells. The IGF2‐Im2/CL2‐Af_EGFR_ complex induced dose‐dependent EGFR degradation in all tested lines (Figure 3k), with the most pronounced effects observed in 5637 and 786‐O cells. These findings demonstrate the versatility of the Type‐I UPTAB platform for membrane protein degradation across diverse cellular contexts.

To address whether prolonged treatment with IGF2‐Im2/CL2‐Af_EGFR_ affects IGF2R expression, we performed WB analysis of IGF2R levels in HeLa and 5637 cells treated with the IGF2‐Im2/CL2‐Af_EGFR_ for 48 h (Figure S10). IGF2R levels remained unchanged compared to untreated controls. This indicates that the UPTAB platform does not induce significant downregulation of the IGF2R, further supporting the sustained efficacy of the system in our study. For the EGFR‑targeted Type‑I UPTAB, we examined the phosphorylation status of key downstream effectors in HeLa cells (Figure S11). Treatment with the degrader (IGF2‑Im2/CL2‑Af_EGFR_) led to a significant reduction in p‑EGFR and p‑AKT levels, whereas treatment with the inhibitor (CL2‑Af_EGFR_ alone) did not. These results indicate that degrader‑driven target degradation can exert more profound effects on downstream signaling than simple blockade of EGFR by the inhibitor alone.

### 2.4 Type‐I UPTAB Mediates In Vivo Protein Degradation and Inhibits Orthotopic Tumor Growth

**FIGURE 4:**
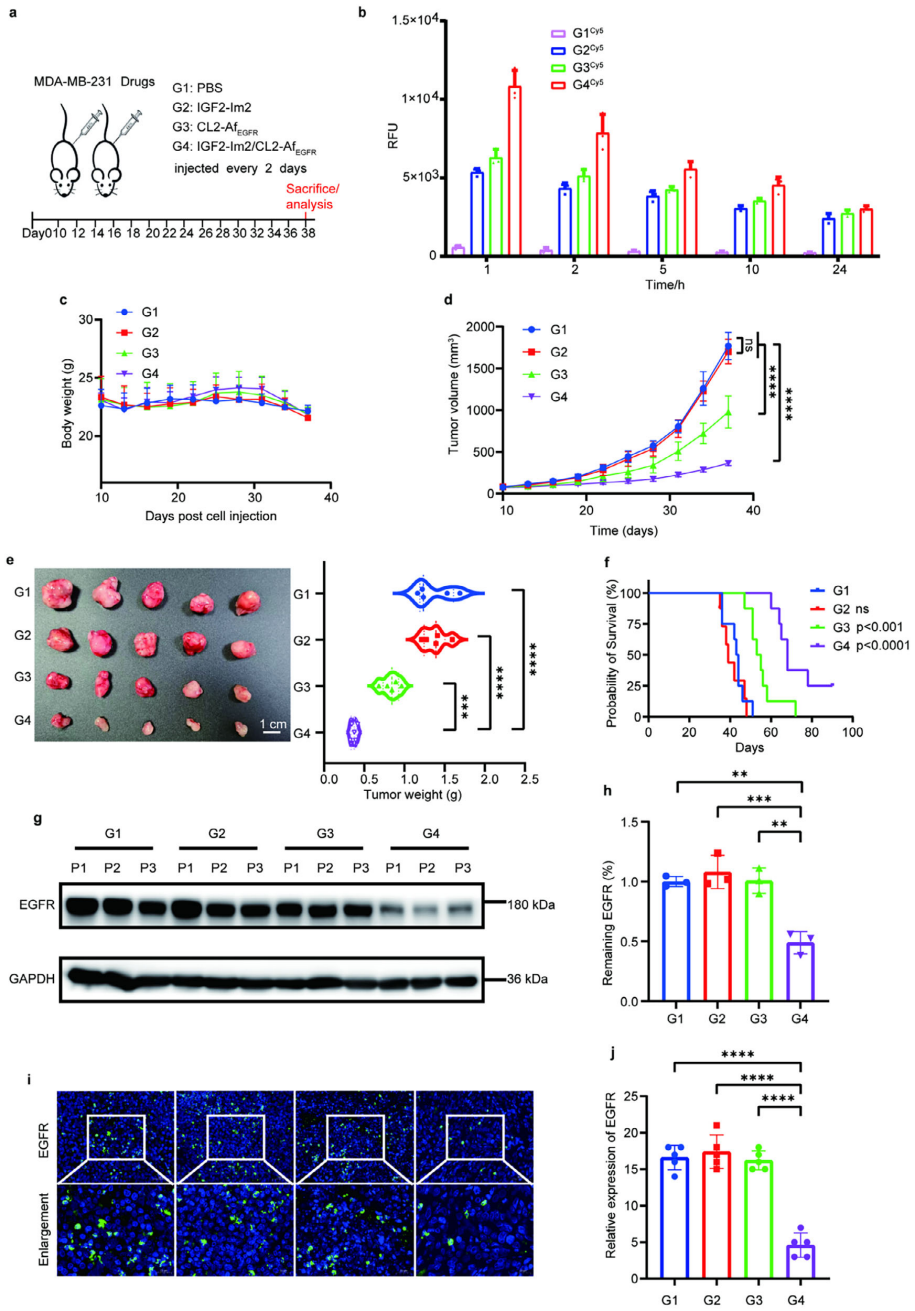
In vivo studies of IGF2‐Im2/CL2‐Af_EGFR_ in mouse models. (a) Schematic illustration of the in vivo experimental timeline. MDA‐MB‐231 cells were orthotopically implanted into the mammary fat pad of mice, followed by intravenous administration of indicated formulations (∼10 mg/kg per protein component). (b) Pharmacokinetic profiles of G1 (Tris‐quenched Cy5‐NHS in PBS), G2 (Cy5 labeled IGF2‐Im2), G3 (Cy5 labeled CL2‐Af_EGFR_), and G4 (Cy5 labeled IGF2‐Im2/CL2‐Af_EGFR_) in mice over 24 h (n = 3 mice per group). (c) Body weight measurements of mice from each treatment group were recorded every 3 days throughout the study (n = 7). (d) Tumor growth curves from day 10 to day 37 post‐implantation (n = 7). (e) Photograph of dissected tumors at day 37 (left) and quantification of tumor weights (right) (n = 5). (f) The probability survival curves of mice in each treatment group (n = 8). (g) WB analysis of EGFR levels in dissected tumor tissues (n = 3). P1‐P3 denote samples from three individual mice per treatment group. GAPDH served as a loading control. (h) Quantification of EGFR protein levels from WB analysis in (g) (n = 3). (i) IF staining of EGFR in MDA‐MB‐231 tumor sections from each treatment group (n = 3). Scale bar = 20 µm. (j) Quantification of EGFR fluorescence intensity from IF images in (i) (n = 3). Data are shown as mean ± SD. Statistical significance was assessed using one‐way ANOVA with Tukey's multiple comparisons test; ns, not significant; ^*^ *p* < 0.05; ^**^ *p* < 0.01; ^***^ *p* < 0.001; ^****^ *p* < 0.0001.

To further assess the therapeutic potential of the Type‐I UPTAB platform in vivo, we first characterized its pharmacokinetic profile using a quantitative fluorescence‐based assay. Cy5‐labeled IGF2‐Im2/CL2‐Af_EGFR_ complex, CL2‐Af_EGFR_, IGF2‐Im2, and Tris‐quenched Cy5‐NHS were administered intravenously. Relative fluorescence units (RFU) were monitored at multiple time points (1, 2, 5, 10, and 24 h) post‐injection, with clearance rates determined by measuring fluorescent intensity in tail blood samples. Among all treatment groups, mice receiving the IGF2‐Im2/CL2‐Af_EGFR_ exhibited the most prominent and sustained fluorescent signal kinetics, indicating enhanced circulatory stability (Figure 4b). In contrast, the Cy5‐NHS group displayed the fastest clearance, consistent with the established principle that molecular size profoundly influences pharmacokinetic behavior.

Having established a favorable pharmacokinetic profile, we next evaluated the antitumor efficacy of the Type‐I UPTAB platform using an orthotopic xenograft model. Balb/c nude mice were implanted with MDA‐MB‐231 cells and randomized into four treatment groups (G1‐G4). When tumor volumes reached approximately 100 mm^3^ (day 10), mice received intravenous injections every two days as follows: G1 (PBS control), G2 (IGF2‐Im2), G3 (CL2‐Af_EGFR_), and G4 (pre‐assembled IGF2‐Im2/CL2‐Af_EGFR_ complex) (Figure 4a). The treatment regimen was well tolerated, with no significant body weight loss or other adverse effects observed in any group (Figure 4c). Mice treated with the IGF2‐Im2/CL2‐Af_EGFR_ complex (G4) exhibited approximately 80% reduction in tumor volume compared to controls (Figure 4d,e). A modest reduction in tumor was also observed in mice treated with CL2‐Af_EGFR_ (G3), consistent with EGFR inhibition via ligand blockade. In contrast, no statistically significant differences in tumor volume or weight were detected between G2 (IGF2‐Im2)‐ and G1 (PBS)‐treated mice, confirming that the IGF2R‐binding module alone lacks therapeutic activity. Mice were sacrificed when tumors in the PBS group (G1) reached approximately 2000 mm^3^, and harvested tumors were processed for further analysis (Figure 4e–j).

To confirm that the observed antitumor effects resulted from TPD, we analyzed EGFR levels in excised tumor tissues by WB. Consistent with our *in* vitro findings, a significant reduction of EGFR levels was observed in the G4 treatment group, whereas G1‐G3 showed no appreciable EGFR degradation (Figure 4g,h). Immunofluorescence (IF) staining of tumor sections further corroborated these results, revealing markedly reduced EGFR signal specifically in tumors from G4‐treated mice (Figure 4i,j). Together, these data confirm successful in vivo EGFR degradation mediated by the Type‐I UPTAB platform. Importantly, the enhanced antitumor efficacy observed in G4 translated into a significant survival benefit. The survival period of tumor‐bearing mice in the G4 group was substantially extended compared to all control groups (G1‐G3) (Figure 4f). Notably, the targeted degradation approach (G4) demonstrated superior efficacy compared to EGFR inhibition alone (G3), underscoring the therapeutic advantage of TPD over conventional inhibition strategies for cancer treatment.

### 2.5 Type‐I UPTAB Enables Modular Customization of Degrader‐Drug Conjugates

Both antibody‐drug conjugates (ADCs) and Type‐I UPTABs rely on internalization and lysosomal trafficking to exert their effects, suggesting that these platforms operate through convergent mechanisms. We therefore hypothesized that conjugating a cytotoxic payload to a nanobody‐based degrader could further enhance the cancer cell‐killing potency of the UPTAB platform. Given that IGF2 contains six cysteine residues amenable to maleimide conjugation, we selected IGF2‐Im2 as the “degrader” module for site‐specific conjugation with the vcMMAE payload. Following assembly with the CL2‐Af_EGFR_ “target” module, the resulting DDC was anticipated to undergo lysosomal trafficking, where cathepsin‐mediated cleavage of the VC linker would release free MMAE, a potent auristatin derivative that inhibits tubulin polymerization [48].

**FIGURE 5:**
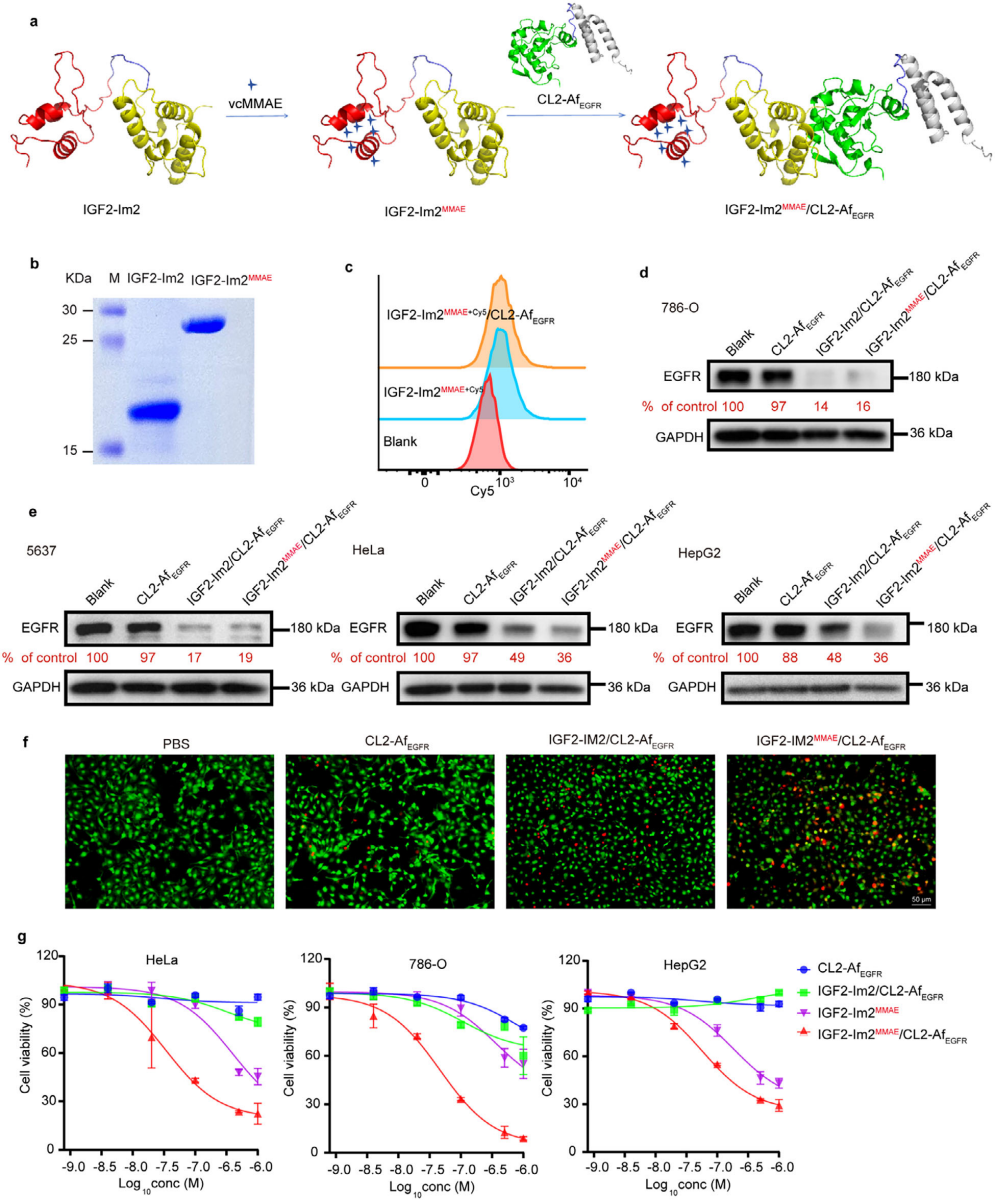
Preparation and characterization of the modular DDC with enhanced antitumor potency. (a) Schematic illustration of DDC synthesis. Maleimide‐modified MMAE was conjugated to IGF2‐Im2, followed by assembly with CL2‐Af_EGFR_ to generate the IGF2‐Im2^MMAE^/CL2‐Af_EGFR_ complex. (b) SDS‐PAGE analysis of purified IGF2‐Im2^MMAE^. Unconjugated IGF2‐Im2 is shown as a molecular weight reference. (c) Flow cytometric analysis of cellular uptake of both Cy5 and MMAE labeled IGF2‐Im2^MMAE+Cy5^ or pre‐assembled with CL2‐Af_EGFR_ (100 nM) in HeLa cells after 6 h incubation. (d,e) WB analysis of EGFR degradation in 786‐O, 5637, HeLa, and HepG2 cells treated with CL2‐Af_EGFR_, IGF2‐Im2/CL2‐Af_EGFR_, or IGF2‐Im2^MMAE^/CL2‐Af_EGFR_ (1000 nM) for 48 h (n = 3 independent experiments). (f) live/dead cell staining images of EGFR‐positive cancer cells following treatment with indicated formulations (500 nM). Green (calcein‐AM): live cells; red (propidium iodide): dead cells. Scale bar = 20 µm. (g) Cytotoxicity of CL2‐Af_EGFR_, IGF2‐Im2/CL2‐Af_EGFR_, IGF2‐Im2^MMAE^ and IGF2‐Im2^MMAE^/CL2‐Af_EGFR_ in EGFR‐positive tumor cell lines (HeLa, 786‐O, and HepG2) after 72 h treatment (n = 3 independent experiments). Cell viability was assessed by standard assays and expressed as a percentage relative to untreated controls. Data are shown as mean ± SD.

Structural modeling with AlphaFold 3 confirmed that the six cysteine residues of IGF2 are surface‐exposed and accessible, potentially allowing simultaneous conjugation with up to six maleimide‐modified vcMMAE molecules (Figure 5a). To construct DDCs, vcMMAE (1.316 kDa) was conjugated to IGF2‐Im2 immobilized on Ni‐NTA agarose for 12 h, followed by removal of unreacted payload. The eluted IGF2‐Im2^MMAE^ was subsequently assembled with CL2‐Af_EGFR_ at a 1:1 molar ratio to generate IGF2‐Im2^MMAE^/CL2‐Af_EGFR_. SDS‐PAGE analysis revealed a single band with reduced mobility compared to unconjugated IGF2‐Im2, consistent with successful conjugation of approximately six vcMMAE molecules (Figure 5b).

We next assessed whether payload conjugation affected the functional properties of the UPTAB platform. Flow cytometric analysis revealed that IGF2R‐mediated endocytosis of IGF2‐Im2 was not impaired by vcMMAE conjugation, nor was cellular uptake of IGF2‐Im2^MMAE^ affected by subsequent assembly with CL2‐Af_EGFR_ (Figure 5c). These results confirm that payload conjugation and complex formation do not compromise the targeting and internalization capacity of the UPTAB system. Having established that DDCs retain efficient cellular uptake, we investigated whether vcMMAE conjugation impacted the degradation efficacy of membrane proteins. WB analysis of EGFR levels was conducted across a panel of five cancer cell lines, including 786‐O, 5637, HeLa, HepG2, and MDA‐MB‐231 cells (Figure 5d,e, and Figure S12). Both IGF2‐Im2^MMAE^/CL2‐Af_EGFR_ (DDC) and IGF2‐Im2/CL2‐Af_EGFR_ (degrader alone) induced significant EGFR degradation in all cell lines tested, whereas CL2‐Af_EGFR_ (inhibitor) showed no degradation activity. Notably, IGF2‐Im2^MMAE^/CL2‐Af_EGFR_ achieved more pronounced EGFR degradation than IGF2‐Im2/CL2‐Af_EGFR_ in HeLa and HepG2 cells, suggesting that Type‐I DDCs offer enhanced cancer cell‐killing efficacy through combined degradation and payload delivery.

Having established that Type‐I UPTAB mediates mono‐targeted degradation, we next sought to determine whether MMAE conjugation further enhances its cytotoxic potency. To compare the cell‐killing potency of DDCs, degraders, and inhibitors, we performed live/dead cell staining and CCK‐8 viability assays. Live/dead staining revealed a marked increase in the proportion of dead cells following treatment with IGF2‐Im2^MMAE^/CL2‐Af_EGFR_ compared to IGF2‐Im2/CL2‐Af_EGFR_ (Figure 5f and Figure S13). Consistent with our degradation data, IGF2‐Im2/CL2‐Af_EGFR_ also induced greater cell death than CL2‐Af_EGFR_. Quantitative CCK‐8 assays further demonstrated dose‐dependent reductions in cell viability across the “degraders” and “DDCs” groups, with IGF2‐Im2^MMAE^/CL2‐Af_EGFR_ exhibiting the most potent anti‐proliferative activity in diverse cancer cell lines (Figure 5g). IGF2‐Im2^MMAE^ alone exhibited only modest cytotoxicity across the tested cell lines (HeLa, 786‐O, and HepG2). In contrast, the DDC (IGF2‐Im2^MMAE^/CL2‐Af_EGFR_) achieved >70% reduction in viability under the same conditions. These results demonstrate that the potent anti‐proliferative activity of the DDC arises from targeted EGFR degradation combined with MMAE delivery, rather than from uptake of the IGF2‐Im2^MMAE^ alone. This dual‐mechanism strategy represents a potential approach for improving the anti‐tumor efficacy of targeted protein degraders. Notably, IGF2‐Im2/CL2‐Af_EGFR_ and CL2‐Af_EGFR_ without MMAE conjugation showed only modest cytotoxicity in 786‐O cells, with the degrader consistently outperforming the inhibitor. Collectively, these results demonstrate that Type‐I DDCs enhance anti‐tumor effects through degrader‐mediated delivery of the payload MMAE into tumor cells, combining the advantages of TPD with the potent cell‐killing activity of chemotherapy. This dual‐mechanism strategy represents a potential approach for improving the anti‐tumor efficacy of targeted protein degraders.

Having established the potent on‑target activity of the Type‑I DDC, we next investigated its selectivity profile to rule out possible off‑target effects. To investigate possible off‑target effects of the Type‑I DDC, we also evaluated the cytotoxicity of the inhibitor, degrader, and DDC (IGF2‐Im2^MMAE^/CL2‐Af_EGFR_) in non‑malignant HEK293 cells. For the DDC format, we assessed viability and observed an IC_50_ shift of >10‑fold between cancer cells and non‑malignant HEK293 cells, supporting a favorable selectivity margin (Figure S14). To further address unintended effects on other membrane proteins, we treated non‑malignant HEK293 cells and MDA‑MB‑231 cells with the inhibitor, degrader, and DDC (1000 nM, 48 h). Using a panel of antibodies against off‑target membrane proteins (including IGF2R, HER2, c‑MET, Sortilin, TfR, and PD‑L1), we did not detect degradation of these proteins following treatment with the EGFR‑targeted DDC (Figure S15), suggesting selectivity for the intended target.

### 2.6 Type‐II UPTAB Enable Simultaneous Dual‐Targeted Degradation and Modular Customization of Type‐II DDCs

**FIGURE 6:**
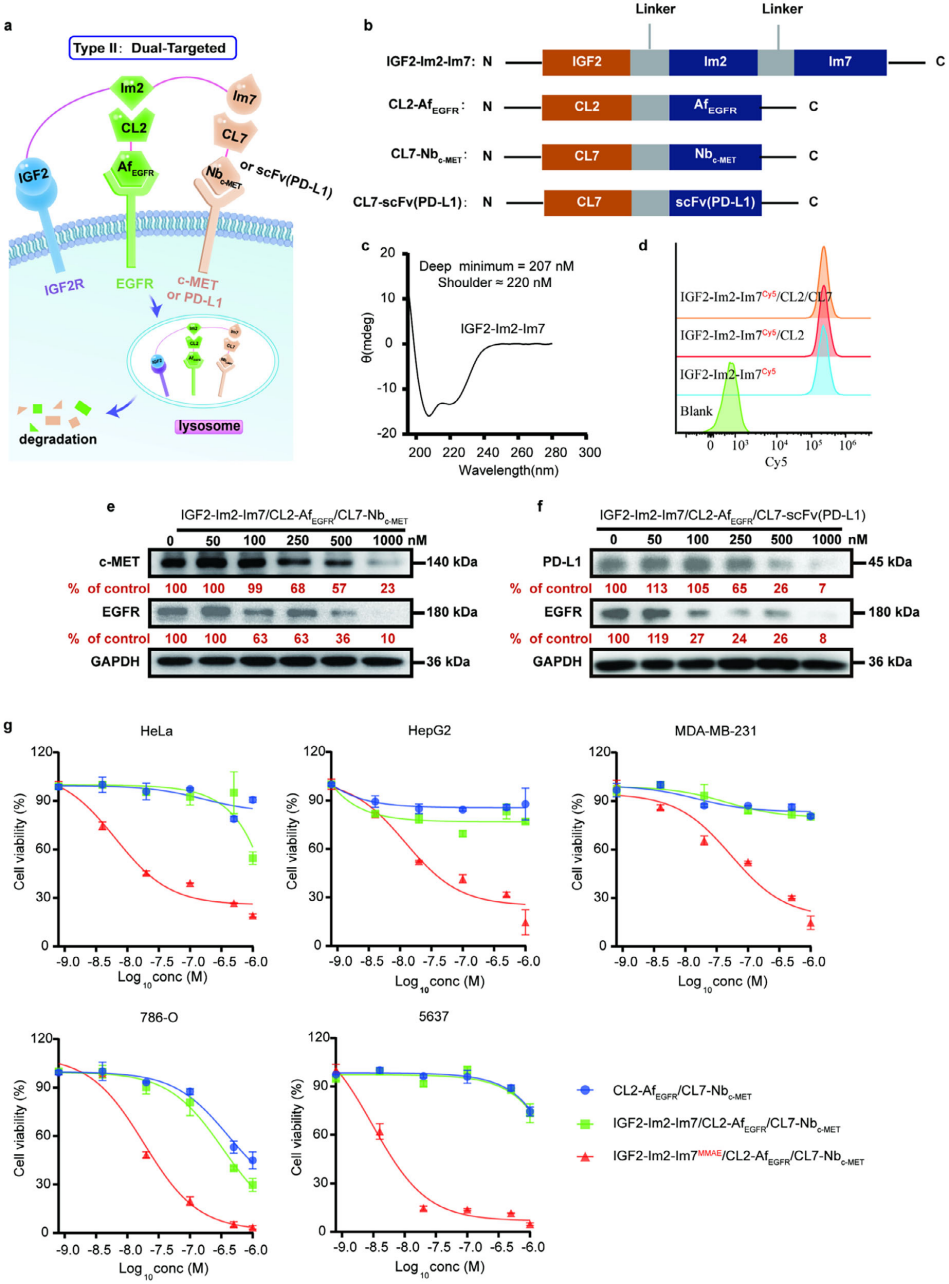
Characterizations and evaluation of Type‐II UPTAB and DDCs. (a) Schematic illustration of dual‐targeted degradation mediated by Type‐II UPTAB. The IGF2‐Im2‐Im7 scaffold simultaneously engages CL2‐fused and CL7‐fused POI binders, enabling concurrent degradation of two membrane proteins. (b) Schematic representation of gene constructs used in this study: the IGF2R‐binding scaffold IGF2‐Im2‐Im7, and POI‐binding modules CL2‐Af_EGFR_, CL7‐Nb_c‐MET_, and CL7‐scFv(PD‐L1). (c) CD spectrum of purified IGF2‐Im2‐Im7 in 10 mM potassium phosphate buffer (pH 7.2). (d) Flow cytometric analysis of cellular uptake of Cy5‐labeled IGF2‐Im2‐Im7 alone or following pre‐assembly with CL2‐Af_EGFR_ or with both CL2‐Af_EGFR_ and CL7‐Nb_c‐MET_ (100 nM each) in HeLa cells after 6 h incubation. CL2 denotes CL2‐Af_EGFR_; CL7 denotes CL7‐Nb_c‐MET_. (e) WB analysis of EGFR and c‐MET levels in MDA‐MB‐231 cells treated with IGF2‐Im2‐Im7 pre‐assembled with CL2‐Af_EGFR_ and CL7‐Nb_c‐MET_ at indicated concentrations (0–1000 nM) for 48 h (n = 3 independent experiments). (f) WB analysis of EGFR and PD‐L1 levels in MDA‐MB‐231 cells treated with IGF2‐Im2‐Im7 pre‐assembled with CL2‐Af_EGFR_ and CL7‐scFv(PD‐L1) at indicated concentrations (0‐1000 nM) for 48 h (n = 3 independent experiments). (g) Cell viability after 72 h treatment with CL2‐Af_EGFR_/CL7‐Nb_c‐MET_ (inhibitors), IGF2‐Im2‐Im7/CL2‐Af_EGFR_/CL7‐Nb_c‐MET_ (degrader alone), and IGF2‐Im2‐Im7^MMAE^/CL2‐Af_EGFR_/CL7‐Nb_c‐MET_ (DDC) in HeLa, HepG2, MDA‐MB‐231, 786‐O, and 5637 cells (n = 3 independent experiments). Cell viability was assessed using standard assays and normalized to untreated controls. Data are shown as mean ± SD.

To expand the versatility of the UPTAB platform for simultaneous dual‐targeted degradation, we designed a Type‐II UPTAB configuration (Figure 6a). Leveraging the orthogonality of the Im2/CL2 and Im7/CL7 pairs, we fused both Im2 and Im7 domains to IGF2, generating the IGF2R‐binding scaffold IGF2‐Im2‐Im7 (Figure 6b). The CL2‐Af_EGFR_, CL7‐Nb_c‐MET_, and CL7‐scFv(PD‐L1) were constructed to enable dual engagement of membrane protein pairs. The predicted structural model of IGF2‐Im2‐Im7 (with IGF2 in red, Im2 in yellow, and Im7 in green) illustrated the rational design of this fusion scaffold, demonstrating the spatial accessibility of the orthogonal Im2 and Im7 domains for simultaneous CL2 and CL7 module assembly (Figure S16). Successful expression of these fusion proteins was confirmed by SDS‐PAGE (Figure S17a). We next characterized the structural integrity of these proteins using CD spectroscopy. The CD spectrum of IGF2‐Im2‐Im7 exhibited characteristic minima at approximately 208 and 222 nm, indicative of a predominantly α‐helical conformation consistent with structural predictions (Figure 6c). Similarly, CD analysis of CL7‐Nb_c‐MET_ revealed a mixed α‐helical/β‐sheet conformation (Figure S18b), in agreement with its predicted structure (Figure S18a). Together, these findings confirmed that the recombinant Im‐fused and CL‐fused proteins preserve the native folding of each constituent domain, a prerequisite for functional activity. To assess complex formation, we performed SEC analysis, which revealed a single, well‐defined peak corresponding to the assembled IGF2‐Im2‐Im7/CL2‐Af_EGFR_/CL7‐Nb_c‐MET_ ternary complex, establishing the structural foundation for dual‐targeted degradation (Figure S17b). Stable complex assembly was further validated by Ni‐NTA pull‐down assays, in which untagged IGF2‐Im2‐Im7 and CL7‐Nb_c‐MET_ co‐eluted with His‐tagged CL2‐Af_EGFR_ in the presence of 200 mM imidazole, confirming specific and stable interactions among all three components (Figure S19). Having established structural integrity, we evaluated the receptor engagement and internalization capacity of the Type‐II UPTAB platform. Flow cytometric analysis of cells treated with Cy5‐labeled IGF2‐Im2‐Im7 or following pre‐assembly with CL2‐Af_EGFR_ or with both CL2‐Af_EGFR_ and CL7‐Nb*_c_*_​‐MET_ revealed that IGF2R‐mediated endocytosis remained unimpaired upon complex formation (Figure 6d). These results confirm that assembly with one or two POI‐binding modules does not compromise cellular uptake, validating the functional capacity of the Type‐II UPTAB platform for simultaneous delivery of dual targets to the lysosomal degradation pathway.

We next evaluated the degradation efficacy of the Type‐II UPTAB platform. Cells were treated with increasing concentrations of IGF2‐Im2‐Im7 pre‐assembled with either CL2‐Af_EGFR_ and CL7‐Nb*_c_*_‐MET_ (for EGFR/c‐MET dual degradation) or CL2‐Af_EGFR_ and CL7‐scFv(PD‐L1) (for EGFR/PD‐L1 dual degradation). WB analysis revealed dose‐dependent reductions in these target protein pairs (Figure 6e,f). For the EGFR and c‐MET combination, maximum degradation efficiencies reached 77% and 90%, respectively. Similarly, treatment with IGF2‐Im2‐Im7/CL2‐Af_EGFR_/CL7‐scFv(PD‐L1) resulted in dose‐dependent degradation of both EGFR and PD‐L1, with maximum efficiencies achieving 92% and 93% for each target. To determine whether the observed degradation of dual‐targets depends specifically on IGF2R‐mediated lysosomal trafficking rather than on crosslinking‐induced effects alone, we performed two approaches. First, we knocked out IGF2R in MDA‐MB‐231 cells using siRNA. IGF2R expression was substantially reduced in siRNA‐treated cells (Figure S20a). Under these conditions, the degradation of EGFR and c‐MET by Type‐II UPTABs was almost completely abrogated (Figure S20b). Second, we utilized A549 cells, which have been reported to be naturally IGF2R‐deficient [20]. Consistent with the knockout data, the Type‐II UPTAB‐mediated degradation was abolished in A549 cells (Figure S20c,d). These results confirm that the degradation activity of the Type‐II UPTABs is strictly dependent on IGF2R‐mediated internalization and is not an artifact of crosslinking. These results directly demonstrate that the IGF2‐Im2‐Im7 scaffold mediates concurrent lysosomal degradation of two membrane proteins, establishing the Type‐II UPTAB as a versatile platform for dual‐targeted protein degradation.

Having established that Type‐II UPTAB mediates dual‐targeted degradation, we next sought to determine whether MMAE conjugation further enhances its cytotoxic potency. To compare the cell‐killing potency of DDCs, degraders, and inhibitors, we performed CCK‐8 viability assays across a panel of cancer cell lines. Dose‐dependent reductions in cell viability were observed for both degrader (IGF2‐Im2‐Im7/CL2‐Af_EGFR_/CL7‐Nb_c‐MET_) and DDC (IGF2‐Im2‐Im7^MMAE^/CL2‐Af_EGFR_/CL7‐Nb_c‐MET_) treatment groups, with the DDC exhibiting the most potent anti‐proliferative activity in diverse cancer cell lines (Figure 6g). In contrast, the inhibitors (CL2‐Af_EGFR_/CL7‐Nb_c‐MET_) and unconjugated degrader showed slight cytotoxicity, with the exception of 786‐O cells, where the degrader alone demonstrated intermediate activity. Collectively, these results demonstrated that Type‐II DDCs enhance cancer cell‐killing effects through degrader‐mediated delivery of the cytotoxic payload MMAE into tumor cells, highlighting the anti‐tumor potential of the conjugated Type‐II UPTAB platform.

### 2.7 Type‐III UPTAB Enable Simultaneous Tri‐Targeted Degradation and Modular Customization of Type‐III DDCs

**FIGURE 7:**
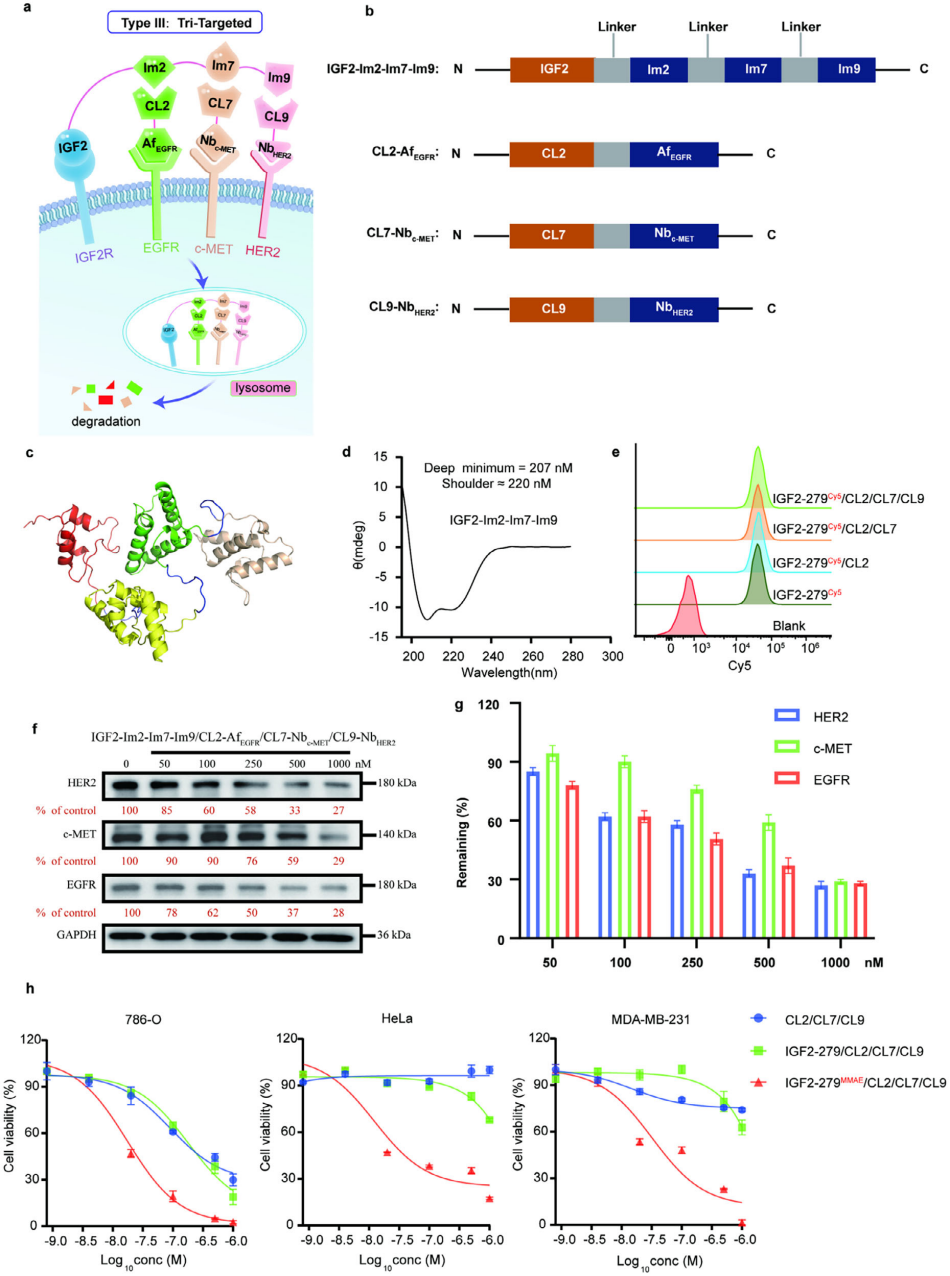
Characterizations and evaluation of Type‐III UPTAB and DDCs. (a) Schematic illustration of tri‐targeted degradation mediated by Type‐III UPTAB. The IGF2‐Im2‐Im7‐Im9 scaffold simultaneously engages CL2‐fused, CL7‐fused, and CL9‐fused POI binders, enabling concurrent degradation of three membrane proteins. (b) Schematic representation of gene constructs used in this study: the IGF2R‐binding scaffold IGF2‐Im2‐Im7‐Im9, and POI‐binding modules CL2‐Af_EGFR_, CL7‐Nb_c‐MET_, and CL9‐Nb_HER2_. (c) AlphaFold3‐predicted structures of IGF2‐Im2‐Im7‐Im9. IGF2 is shown in red, Im2 in yellow, Im7 in green, and Im9 in gray, with GS linkers highlighted in blue. (d) CD spectrum of purified IGF2‐Im2‐Im7‐Im9 in 10 mM potassium phosphate buffer (pH 7.2). (e) Flow cytometric analysis of cellular uptake of Cy5‐labeled IGF2‐Im2‐Im7‐Im9 alone or following pre‐assembly with CL2‐Af_EGFR_ alone, CL2‐Af_EGFR_/CL7‐Nb_c‐MET_, or CL2‐Af_EGFR_/CL7‐Nb_c‐MET_/CL9‐Nb_HER2_ (100 nM) in HeLa cells after 6 h incubation. CL2 denotes CL2‐Af_EGFR_; CL7 denotes CL7‐Nb_c‐MET_; CL9 denotes CL9‐Nb_HER2_. (f) WB analysis of EGFR, c‐MET, and HER2 levels in MDA‐MB‐231 cells treated with IGF2‐Im2‐Im7‐Im9 pre‐assembled with CL2‐Af_EGFR_, CL7‐Nb_c‐MET_, and CL9‐Nb_HER2_ at indicated concentrations (0–1000 nM) for 48 h (n = 3 independent experiments). (g) Quantitative analysis of EGFR, c‐MET, and HER2 levels in (f) (n = 3 independent experiments). Band intensities were normalized and expressed as a percentage relative to untreated controls. (h) Cell viability after 72 h treatment with CL2‐Af_EGFR_/CL7‐Nb_c‐MET_/CL9‐Nb_HER2_ (inhibitors), IGF2‐Im2‐Im7‐Im9/CL2‐Af_EGFR_/CL7‐Nb_c‐MET_/CL9‐Nb_HER2_ (degrader alone), and IGF2‐Im2‐Im7‐Im9^MMAE^/CL2‐Af_EGFR_/CL7‐Nb_c‐MET_/CL9‐Nb_HER2_ (DDC) in 786‐O, HeLa, MDA‐MB‐231 (n = 3 independent experiments). IGF2‐279 denotes IGF2‐Im2‐Im7‐Im9. Cell viability was assessed using standard assays and normalized to untreated controls. Data are shown as mean ± SD.

To further expand the versatility of the UPTAB platform, we designed Type‐III UPTAB configurations capable of simultaneous degradation of three membrane proteins (Figure 7a). Leveraging the orthogonality of the Im2/CL2, Im7/CL7, and Im9/CL9 pairs, we fused Im2, Im7, and Im9 domains to IGF2, generating the multivalent IGF2R‐binding scaffold IGF2‐Im2‐Im7‐Im9 (Figure 7b). CL2‐Af_EGFR_, CL7‐Nb*_c_*_​‐MET_, and CL9‐Nb_HER2_ (HER2‐targeting) were also constructed to enable multivalent engagement. The predicted structural model of IGF2‐Im2‐Im7‐Im9 (with IGF2 in red, Im2 in yellow, Im7 in green, and Im9 in gray) illustrated the rational design of this fusion scaffold, demonstrating the spatial accessibility of the orthogonal Im2, Im7, and Im9 domains for simultaneous assembly with their CL‐fused modules (Figure 7c).

Successful expression of these fusion proteins was confirmed by SDS‐PAGE (Figure S21a). We next characterized the structural integrity of these purified proteins using CD spectroscopy. The CD spectrum of IGF2‐Im2‐Im7‐Im9 exhibited characteristic minima at approximately 208 nm and 222 nm, indicative of a predominantly α‐helical conformation consistent with structural predictions (Figure 7d). Similarly, CD analysis of CL9‐Nb_HER2_ revealed a mixed α‐helical/β‐sheet conformation (Figure S22b), in agreement with its predicted structure (Figure S22a). Together, these findings confirmed that the recombinant Im‐fused and CL‐fused proteins preserve the native folding of each constituent domain, a prerequisite for functional activity. To assess complex formation, we performed SEC analysis, which revealed a single, well‐defined peak corresponding to the assembled IGF2‐Im2‐Im7‐Im9/CL2‐Af_EGFR_/CL7‐Nb_c‐MET_/CL9‐Nb_HER2_ complex, establishing the structural foundation for tri‐targeted degradation (Figure S21b). Stable complex assembly was further validated by Ni‐NTA pull‐down assays, in which untagged IGF2‐Im2‐Im7‐Im9, CL7‐Nb_c‐MET_, and CL9‐Nb_HER2_ co‐eluted with His‐tagged CL2‐Af_EGFR_ in the presence of 200 mM imidazole, confirming specific and stable interactions among all four components (Figure S23).

We next evaluated the receptor engagement and internalization capacity of the Type‐III UPTAB platform. Flow cytometric analysis of cells treated with Cy5‐labeled IGF2‐Im2‐Im7‐Im9 or following pre‐assembly with CL2‐Af_EGFR_, CL2‐Af_EGFR_/CL7‐Nb_c‐MET_ or CL2‐Af_EGFR_/CL7‐Nb_C‐MET_/CL9‐Nb_HER2_ revealed that IGF2R‐mediated endocytosis remained unimpaired upon complex formation (Figure 7e). These results confirmed that assembly with up to three POI‐binding modules does not compromise cellular uptake, validating the functional capacity of the Type‐III UPTAB platform for simultaneous delivery of three targets to the lysosomal degradation pathway. Having confirmed successful internalization, we next evaluated the degradation efficacy of the Type‐III UPTAB platform. Cells were treated with increasing concentrations of IGF2‐Im2‐Im7‐Im9 pre‐assembled with CL2‐Af_EGFR_/CL7‐Nb*_c_*_‐MET_/CL9‐Nb_HER2_ (for EGFR/c‐MET/HER2 triple degradation). WB analysis revealed the degradation of EGFR/c‐MET/HER2 in a dose‐dependent manner (Figure 7f,g). All maximum degradation efficiencies exceeded 70% for EGFR, c‐MET, and HER2, demonstrating that the IGF2‐Im2‐Im7‐Im9 scaffold mediates concurrent lysosomal degradation of three membrane proteins. To determine whether the observed degradation of tril‐targets depends specifically on IGF2R‐mediated lysosomal trafficking rather than on crosslinking‐induced effects alone, the Type‐III UPTAB was evaluated in IGF2R‐deficient (MDA‐MB‐231 KO and A549) cells. The degradation of HER2, EGFR, and c‐MET by Type‐III UPTABs was almost completely abrogated, confirming that the degradation activity of the Type‐III UPTABs is strictly dependent on IGF2R‐mediated internalization and is not an artifact of crosslinking (Figure S24). These results established the Type‐III UPTAB as a versatile platform for tri‐targeted degradation.

Finally, we sought to determine whether MMAE conjugation further enhances its cytotoxic potency. To compare the cell‐killing potency of DDCs, degraders, and inhibitors, we performed CCK‐8 viability assays across a panel of cancer cell lines. Dose‐dependent reductions in cell viability were observed for both degrader (IGF2‐Im2‐Im7‐Im9/CL2‐Af_EGFR_/CL7‐Nb*_c_*_​‐MET_/CL9‐Nb_HER2_) and DDC (IGF2‐Im2‐Im7‐Im9^MMAE^/CL2‐Af_EGFR_/CL7‐Nb*_c_*_‐MET_/CL9‐Nb_HER2_) treatment groups, with the DDC exhibiting the most potent anti‐proliferative activity in diverse cancer cell lines (Figure 7h and Figure S25). In contrast, the inhibitor cocktail (CL2‐Af_EGFR_/CL7‐Nb*_c_*_‐MET_/CL9‐Nb_HER2_) and unconjugated degrader showed slight cytotoxicity, with the exception of 786‐O cells. Collectively, these results demonstrate that Type‐III DDCs enhance cancer cell‐killing effects through degrader‐mediated delivery of the cytotoxic payload MMAE into tumor cells, highlighting the anti‐tumor potential of conjugated type‐III UPTAB.

## 3 Conclusion

In this study, we developed a modular and versatile “plug‐and‐play” UPTAB platform for targeted degradation of membrane proteins through the lysosomal pathway. By leveraging ultrahigh‐affinity orthogonal Im/CL protein pairs, we engineered three UPTAB configurations capable of mono‐targeted (Type‐I), dual‐targeted (Type‐II), and tri‐targeted (Type‐III) degradation. This modular system enables rapid customization of degraders by simply mixing Im‐fused LTR‐binding scaffolds with CL‐fused POI‐binding modules, bypassing the need for direct fusions re‐engineering. We demonstrated that Type‐I UPTABs mediate efficient and selective degradation of diverse targets, including EGFR and PD‐L1, achieving over 90% target elimination *in* *vitro* and up to 80% tumor growth inhibition in an orthotopic breast cancer xenograft model. The platform exhibited superior expression yields in E. coli compared to direct fusion constructs, and optimization of linker length identified optimal configurations for maximal degradation activity. Mechanistic studies confirmed that UPTABs direct target proteins to lysosomes via LTR‐mediated endocytosis, with degradation abrogated by lysosomal inhibitors.

Importantly, we extended the UPTAB platform to generate DDCs by site‐specific conjugation of the cytotoxic payload MMAE to the degrader module (IGF2), bypassing the need for additional protein mutations to introduce cysteine residues. DDCs retained full degradation capacity while acquiring potent cytotoxic activity through payload delivery, resulting in substantially enhanced anti‐proliferative effects across multiple cancer cell lines. This dual‐mechanism strategy combines the advantages of TPD with the potent cell‐killing activity of chemotherapy. The modularity of the UPTAB platform enabled seamless extension to dual‐ and tri‐targeted degradation. Type‐II UPTABs simultaneously degraded EGFR/c‐MET and EGFR/PD‐L1 pairs with high efficiency, while Type‐III UPTABs achieved concurrent degradation of EGFR, c‐MET, and HER2, with maximum degradation efficiencies exceeding 70% for all three targets. These multivalent configurations address the critical need for strategies that can target the synergistic networks of membrane proteins driving tumor progression and therapy resistance.

In summary, the UPTAB platform combines modular multivalent design, high degradation efficiency, and excellent bioconjugation capability to serve as a versatile tool for membrane protein degradation. The ability to rapidly generate mono‐, dual‐, and tri‐targeted degraders, as well as DDCs with enhanced cancer cell‐killing potency, expands the toolkit for TPD and offers a candidate approach for developing next‐generation therapeutics.

## 4 Experimental Section

Materials can be found in the Supporting Information.

### 4.1 Cell Culture

HeLa, MDA‐MB‐231, 786‐O, HepG2, and 5637 cells were cultured in complete growth medium (DMEM supplemented with 10% FBS and 1% penicillin/streptomycin) under 5% CO_2_ at 37°C. K562 suspension cells were cultured in complete growth medium (RPMI 1640 supplemented with 10% FBS and 1% penicillin/streptomycin). HeLa, MDA‐MB‐231,786‐O, HepG2, K562, and 5637 cells were obtained from the American Tissue Culture Collection (ATCC).

### 4.2 Plasmid Construction and Protein Expression

All expression plasmids were constructed by cloning synthetic DNA sequences encoding the target proteins with an N‐terminal His tag into the pET23a vector, and all constructs were verified by DNA sequencing. For protein expression, plasmids were transformed into chemically competent E. coli BL21(DE3) or Rosetta pLysS cells. Briefly, 1–5 µL of plasmid was gently mixed with thawed competent cells and incubated on ice for 30 min. Following heat shock at 42°C for 45 s, 200 µL of NZY medium was added, and cells were recovered at 37°C for 1 h with shaking. Transformed cells were plated on LB agar containing ampicillin (200 µg/mL) and incubated at 37°C for 12 h. Single colonies were selected for subsequent protein expression. Cell pellets were resuspended in PBS (10 mL per gram of wet cell weight) supplemented with 1% protease inhibitor and lysed by high‐pressure homogenization on ice. An additional 0.1% protease inhibitor was added post‐lysis. Lysates were clarified by centrifugation at 12 000 × g for 30 min at 4°C. The supernatant was incubated with Ni‐NTA resin in a gravity column for 2 h at 4°C with gentle rotation. The resin was washed, and bound proteins were eluted with an imidazole gradient in elution buffer (20 mM sodium phosphate, 500 mM NaCl, pH 7.4). Fractions containing the target protein were identified by SDS‐PAGE, pooled, and concentrated to 3–20 mg/mL using ultrafiltration devices. Glycerol was added to a final concentration of 10%, and proteins were stored at −80°C.

### 4.3 CD

Far‐ultraviolet (far‐UV) CD spectra were recorded from 260 to 195 nm using a spectropolarimeter with a temperature‐controlled cuvette. Protein samples (0.2–1.0 mg/mL in PBS, pH 7.4) were analyzed in a 0.2 mm quartz cuvette at 25°C. Spectra were collected with a 0.2 nm resolution, 2 nm bandwidth, and 50 nm/min scanning speed. CD intensity was expressed as molar ellipticity.

### 4.4 SEC

Proteins purified by Ni‐NTA affinity chromatography were subjected to SEC for secondary purification and analysis. The SEC system and an appropriate size‐exclusion column (selected based on the target protein's molecular weight) were equilibrated sequentially with ultrapure water and protein storage buffer. Protein samples at concentrations of at least 3 mg/mL were loaded via the sample loop. Following separation, eluted fractions were collected. Chromatograms displaying multiple peaks were further analyzed to determine the protein composition of corresponding fractions. Fractions corresponding to the target peak were pooled, concentrated, and protein concentration was determined using a microplate reader.

### 4.5 Ni‐NTA Based Pull‐Down Assays

To validate protein pair interactions, equimolar amounts of each protein were incubated in PBS buffer at room temperature for 10 min to allow sufficient binding. Ni‐NTA beads (200 µL) were then added, and the mixture was incubated with gentle rotation at 4°C for 1 h. Following incubation, the supernatant was removed by centrifugation, and the beads were washed sequentially with PBS containing 10, 50, and 200 mM imidazole. Protein samples from each wash step were collected. For analysis, 40 µL of each sample was mixed with 10 µL of 5× SDS‐PAGE loading buffer and heated at 100°C for 5 min in a metal bath to denature proteins. Samples were then resolved by SDS‐PAGE, visualized by Coomassie Brilliant Blue staining followed by destaining, and imaged using a gel documentation system.

### 4.6 Fluorescent Labeling of Proteins

For fluorescent labeling, 1 mg of protein was conjugated with 50 µg of Cy5‐NHS. Briefly, 100 µL of protein solution (1 mg/mL in PBS) was diluted into 400 µL of Na_2_CO_3_ buffer (500 mM, pH 9.0). Cy5‐NHS (6 µL, 1 mM) was then added, and the reaction mixture was incubated at 4°C overnight in the dark. The reaction was quenched by the addition of Tris‐HCl buffer (pH 8.0). Free dye was removed by buffer exchange with PBS containing 2% DMSO using ultrafiltration centrifugation, with all steps performed under strict light protection. For the control experiment, an equivalent amount of Cy5‐NHS was quenched with Tris‐HCl buffer and used directly as the dye‐only control.

### 4.7 Synthesis of Modular DDCs

For MMAE conjugation, IGF2‐Im2 was reacted with maleimide‐modified vcMMAE at a 1:10 molar ratio at 4°C for 12 h. Unconjugated vcMMAE was removed by washing with PBS using ultrafiltration, yielding purified IGF2‐Im2MMAE. The resulting DDC was then assembled by mixing IGF2‐Im2^MMAE^ with CL2‐Af_EGFR_ at a 1:1 molar ratio and incubating at room temperature for 10 min. Other DDCs were generated analogously by combining other Im‐fused and CL‐fused protein pairs using the same strategy.

### 4.8 Cell Cytotoxicity Assay

Cells were seeded in 96‐well plates at densities ranging from 2000 to 5000 cells per well, according to their respective growth rates, and allowed to adhere overnight. The medium was then replaced with fresh complete medium containing protein drugs at the indicated concentrations, and cells were incubated for 72 h. After treatment, the medium was carefully removed. Control groups included cells treated with PBS in complete medium, while blank controls consisted of medium containing 10% CCK‐8 reagent without cells. Each well received 100 µL of fresh complete medium supplemented with 10% CCK‐8 reagent, and plates were incubated at 37°C for 30 min to 2 h, protected from light. The reaction was terminated when the solution developed an orange‐yellow color, typically corresponding to an absorbance value of 0.8–1.0 at 450 nm. If color development was insufficient, incubation time was extended accordingly. Absorbance was measured at 450 nm using a microplate reader, and cell viability and inhibition rates were calculated relative to control groups.

### 4.9 Flow Cytometry Analysis

Cells were seeded in 6‐well plates at 70%–80% confluency and cultured overnight at 37°C to allow full adherence. The medium was then replaced with fresh complete medium containing 50 nM Cy5‐labeled proteins targeting specific receptors, and cells were incubated for 2 h at 37°C in the dark. Control groups received complete medium containing PBS. After incubation, cells were washed three times with PBS to remove unbound Cy5‐labeled proteins. Cells were then detached with trypsin, and the reaction was terminated by adding complete medium. The cell suspension was collected by centrifugation at 800 rpm, washed 2–3 times with PBS to remove residual trypsin and medium, and finally resuspended in 200 µL PBS for flow cytometric analysis. All steps were performed under light‐protected conditions.

### 4.10 Confocal Laser Scanning Microscopy

HeLa cells were seeded on glass‐bottom confocal dishes and cultured until they reached 40%–50% confluence. After two washes with PBS, cells were treated with fresh complete medium containing IGF2‐Im2/CL2‐Af_EGFR_^Cy5^. Control groups included cells treated with IGF2‐Im2, Cy5‐labeled CL2‐Af_EGFR_, or PBS alone. Incubation was carried out at 37°C for 12 h. Following treatment, cells were gently rinsed 2–3 times with PBS and subsequently stained with Hoechst 33342 and LysoTracker Green. Live‐cell confocal imaging was performed immediately after staining.

### 4.11 siRNA Knockdown

HeLa or MDA‐MB‐231 cells (100 000 cells per well in a 6‐well plate) were transfected with 80 pmoles of siRNA (PAIVIBIO) and SweTransRNA transfection reagent according to the manufacturer's specifications.

### 4.12 WB Analysis

Cells were seeded in 6‐well plates and cultured overnight at 37°C until reaching 70%–80% confluence. Proteins were diluted to the indicated concentrations in complete growth medium and gently mixed before addition to cells. Cells were treated with proteins at specified concentrations and durations according to experimental designs. For inhibitor studies, cells were pretreated with lysosomal inhibitors Baf or CQ, followed by 24 h co‐incubation with IGF2‐Im2/CL2‐Af_EGFR_. After treatment, cells were lysed on ice for 20 min using 150–200 µL of RIPA lysis buffer (neutral pH), detached by repeated pipetting, and supernatants were collected following centrifugation. Protein concentrations were determined by BCA assay. Equal amounts of protein lysates were separated by SDS‐PAGE and transferred onto PVDF membranes at 90 V under ice‐cold conditions. Membranes were blocked with 5% non‐fat milk in TBST for 30 min to 2 h at room temperature with gentle agitation, then washed with TBST. Membranes were incubated with appropriate primary antibodies overnight at 4°C, washed three times with TBST, and incubated with HRP‐conjugated secondary antibodies for 2 h at room temperature. After final washes, blots were developed with ECL substrate and visualized using a chemiluminescence imaging system. Band intensities were quantified using ImageJ software.

### 4.13 Live/Dead Cell Viability Assay

786O cells were plated into 35 mm Petri dishes containing 1 mL DMEM culture media supplemented with 10% FBS and 1% penicillin/streptomycin, and incubated at 37°C for 24 h. After treating the cells with 500 nM CL2‐Af_EGFR_, IGF2‐Im2/ CL2‐Af_EGFR_, and IGF2‐Im2^MMAE^/CL2‐Af_EGFR_ for 48 h, the cells were stained with Calcein/PI Cell Viability Assay Kit according to the manufacturer's protocol. Cells were then incubated for another 15 min at 37°C. Next, cells were washed with 1x PBS buffer and imaged using a confocal microscope. The green fluorescence of calcein AM was excited at 488 nm and detected with a 500–550 nm bandpass filter. Red fluorescence of PI was excited at 633 nm and detected with a 660–710 nm bandpass filter.

### 4.14 In Vivo Experimental Investigation

#### 4.14.1 General Information

Female BALB/c nude mice (6–8 weeks, 18–20 g) were provided by Beijing Vital River Laboratory Animal Technology Co., Ltd. All animals were acclimated to the environment and maintained under controlled conditions: temperature (23°C ± 2°C), humidity (55% ± 5%), and light (12 h light/dark cycle). All the animals were treated according to the Laboratory Animal Care and Use Guidelines of Huazhong University of Science and Technology (Wuhan, China). All efforts were made to minimize animal suffering and to reduce the number of animals used.

#### 4.14.2 Pharmacokinetics Study

IGF2‐Im2 and CL2‐Af_EGFR_ were labeled with Cy5‐NHS according to the above procedure, namely as IGF2‐Im2^Cy5^ and CL2‐Af_EGFR_^Cy5^, respectively. Mice were treated with 10 mg/kg IGF2‐Im2^Cy5^ (0.5 µmol/kg), 12.5 mg/kg CL2‐Af_EGFR_^Cy5^ (0.5 µmol/kg), 10 mg/kg IGF2‐Im2^Cy5^ + 12.5 mg/kg CL2‐Af_EGFR_^Cy5^ or 168 µg/kg Tris quenched Cy5‐NHS (0.25 µmol/kg) through intravenous injection (3 mice for IGF2‐Im2^Cy5^, 3 mice for CL2‐Af_EGFR_^Cy5^, 3 mice for IGF2‐Im2^Cy5^ + CL2‐Af_EGFR_^Cy5^ and 3 mice for Cy5 group). At the indicated time (1 h, 5 h, 10 h, 24 h), blood was taken from the tail. 10 µL blood was further added to 20 µL PBS‐EDTA (10 mM) to avoid blood coagulation. Then, 25 µL diluted blood was transferred to a 96‐well plate to measure Cy5 fluorescent intensity.

#### 4.14.3 In Vivo EGFR Degradation Study

After a 1‐week acclimation period, MDA‐MB‐231 cells were orthotopically implanted into the mammary fat pad of mice, followed by intravenous administration of indicated formulations. When the tumor volume reached approximately 100 mm^3^, the mice were randomly divided into 4 groups (7 mice per group). Intravenous injections of the following samples were administered every two days, based on molar mass ratio: (1) PBS (control group), (2) IGF2‐Im2 (10 mg/kg, 0.5 µmol/kg), (3) CL2‐Af_EGFR_ (12.5 mg/kg, 0.5 µmol/kg), (4) IGF2‐Im2 (10 mg/kg) + CL2‐Af_EGFR_ (12.5 mg/kg). Before each injection, the body weight and tumor volume of the mice were recorded. Drug injections were performed every two days, and the mouse body weight and tumor volume were measured every three days after the first injection. Tumor volume was calculated using a digital caliper according to the formula: V = L × W^2^ × 0.5, where V: volume, L: length, and W: width. All mice were euthanized when the tumor volume reached approximately 2000 mm^3^, and tumors were collected to measure their exact weight. Tumor tissues from different treatment groups were washed with PBS and homogenized in RIPA buffer containing 1 mM protease inhibitor (Beyotime). The samples were incubated on ice for 20 min, then centrifuged at 12 000 rpm and 4°C for 10 min to remove tissue or cell debris. Equal amounts of supernatant were subjected to WB analysis.

#### 4.14.4 Immunofluorescence Experiments

Tissue samples were fixed in 4% paraformaldehyde to preserve antigenicity. Following fixation, the tissues were sectioned and incubated with specific primary antibodies to allow for antigen binding. The resulting antigen‐antibody complexes were then visualized using a chromogenic detection system. The stained sections were subsequently examined to assess the localization and expression levels of the target antigen.

### 4.15 Statistical Analysis

For all data shown, stated values represent the mean ± S.D. of at least three independent experiments (unless otherwise stated). Significant differences between the control and experimental groups were analyzed using Student's t‐test or one‐way ANOVA analysis. GraphPad Prism 9.0 was used for data statistics. (^****^) *p* < 0.0001, (^***^) *p* < 0.001, (^**^) *p* < 0.01, and (^*^) *p* < 0.05 were considered statistically significant differences.

## Ethics Statement

All the animals care, and experimental procedures were conducted in accordance with relevant guidelines and regulations, and were approved by the Ethics Committee of Tongji Medical College, Huazhong University of Science and Technology (Wuhan, China) ([2025] IACUC Number: 4897).

## Conflicts of Interest

The regents of Hubei University have one patent pending for customizing multivalent Degraders, for which W. M and M.Z. are inventors. The remaining authors declare no competing interests.

## Supporting information




**Supporting File**: advs75658‐sup‐0001‐SuppMat.docx.

## Data Availability

The data that support the findings of this study are available from the corresponding author upon reasonable request.
