# Supplementary material for: A Plug‐and‐Play Platform for Customizing Multivalent Degraders and Degrader‐Drug Conjugates
Source: Adv Sci (Weinh). 2026 May 15;13(44):e75658. doi: 10.1002/advs.75658 (PMC13335768; doi:10.1002/advs.75658)
Supplement: Supplementary file 1 [file ADVS-13-e75658-s001.docx]

A Plug-and-Play Platform for Customizing Multivalent Degraders and Degrader-Drug Conjugates

Mengqing Zhao^[a]+^, Yan Deng^[b]+^, Jianjian Han^[a]^, Yong Xie^[a]^, Kai Bao^[a]^, Lixin Ma*^[a]^, Lilong Liu*^[c]^, Wuxiang Mao*^[a]^

[a]	M. Zhao^+^, J. Han, Y. Xie, Prof. K. Bao, Prof. L. Ma, Prof. W. Mao
State Key Laboratory of Biocatalysis and Enzyme Engineering, Hubei Province Key Laboratory of industrial Biotechnology, School of Life Sciences

Hubei University

Wuhan 430062, China

E-mail: malixing@hubu.edu.cn (L.X. Ma); wxmao@hubu.edu.cn (W.X. Mao leading contact)

[b]	Y. Deng^+^

Department of Clinical Laboratory

Union Hospital, Tongji Medical College, Huazhong University of Science and Technology

Wuhan 430022, China

[c]	Dr. L. Liu

Department of Urology

Tongji Hospital, Tongji Medical College, Huazhong University of Science and Technology

Wuhan 430022, China

E-mail: liulilong@hust.edu.cn

[+] These authors contributed equally to this work.

**Table of Contents**

**Section 1 Experimental materials ……………………………………………………………P3**

**Section 2 Supplementary Table………………………………………………………………P3**

**Section 3 Supplementary Figures………………………………………………..………P4-P16**

**Section 4 Amino acid sequence of proteins…………………………………….………P16-P18**

**Section 5 Appended WB images……………………………….………………..………P19-P23**

**Section 1 Experimental materials**

All DNA primers for plasmid construction were synthesized by Sangon Biotech (Shanghai, China) and verified by DNA sequencing. The host strain E. coli. BL21(DE3) cells were purchased from Shanghai Weidi Biotechnology company (China). RPMI 1640 (Cellmax), DMEM (Hyclone) penicillin/streptomycin (Salorbio) and Fetal Bovine Serum (FBS) were purchased from authorized commercial vendors. Cyanine 5-NHS Ester was purchased from Beijing OKeanos Tech (China). LysoTrackerTM Green DND-26 and Hoechst 33342 were purchased from Invitrogen. Tris-Tricine-SDS-PAGE gel preparation kit was purchased from Salorbio (Catalog No: P1320). Live and dead cell double staining kit was purchase from Abbkine (Catalog No: KTA1001). Ni-NTA agarose beads (Qiagen), CCK8 solution (Vazyme, China), chloroquine (Sigma-Aldrich), Bafilomycin A1 (MedChemExpress), vcMMAE (MedChemExpress) were purchased from the indicated suppliers. All other materials and reagents were purchased from commercial sources and used as received, unless stated otherwise. All antibodies used in this study are from commercial sources, and listed in Section 2.

**Section 2 Supplementary table**

**Antibodies used in the current work**

| **Antibodies** | **Source** | **Catalog** | **Dilution** |
| --- | --- | --- | --- |
| GAPDH Monoclonal antibody | Proteintech | 60004-1-Ig | 1:10000 |
| EGFR Monoclonal antibody | Proteintech | 66455-1-Ig | 1:10000 |
| PD-L1/CD274 Monoclonal antibody | Proteintech | 66248-1-Ig | 1:1000 |
| HER2/ErbB2 Polyclonal antibody | Proteintech | 18299-1-AP | 1:5000 |
| c-MET(Cytoplasmic) Polyclonal antibody | Proteintech | 25869-1-AP | 1:1000 |
| Multi-rAb HRP-Goat Anti-Mouse Recombinant Secondary Antibody (H+L) | Proteintech | RGAM001 | 1:10000 |
| HRP-conjugated Goat anti-Rabbit IgG (H+L) | ABclonal | AS014 | 1:5000 |

**Section 3 Supplementary Figures**

**Figure S1.** Characterization of recombination proteins in the Type I UPTAB. SDS-PAGE analysis of bacterially expressed and purified proteins including Im2-fused LTR binding domains and CL2-fused POI binding domains by Coomassie brilliant blue (CBB).

**Figure S2.** Flow cytometry analysis validates the endocytic efficiency of IGF2-Im2 in diverse cancer cell lines. (**a**) Flow cytometric analysis of IGF2-Im2^Cy5^ uptake in MDA-MB-231 breast cancer cells. Cells were incubated with IGF2-Im2^Cy5^ (100 nM) for 6 h at 37 °C, and fluorescence intensity was measured by flow cytometry. (**b)** Flow cytometric analysis of IGF2-Im2^Cy5^ uptake in K562 (chronic myeloid leukemia cells) under the same conditions.

**Figure S3.** Structural prediction and CD spectroscopy analysis of CL2-GFP. (**a**) Structure of CL2-GFP binders predicted by AlphaFold3. The pink domain represents CL2, while the green domain represents GFP. The GS linker is labelled in blue. (**b**) CD spectrum of purified CL2-GFP in 10 mM potassium phosphate buffer (pH 7.2).

**Figure S4**. Flow cytometry analysis of uptake of IGF2-Im2, CL2-GFP and assembled IGF2-Im2/CL2-GFP. (**a**) Cellular uptake of IGF2-Im2 (0-1000 nM) following pre-assembly with CL2-GFP (0-1000 nM) for 4 h in HeLa cells. Unpaired IGF2-Im2 (50 nM) and CL2-GFP (50 nM) alone served as negative controls. (**b**) Time-dependent cellular uptake of pre-assembled IGF2-Im2 with CL2-GFP (1000 nM each) over 0-12 h in HeLa cells, analyzed by flow cytometry.

**Figure S5.** Structural prediction and CD spectroscopy analysis of CL2-fused POI binding domains. (**a**) AlphaFold3-predicted structures of CL2-Af_EGFR_ (left) and CL2-scFv(PD-L1) (right). The CL2 domain is shown in red, the POI-binding domains (Af_EGFR_ or scFv(PD-L1)) in yellow, and GS linkers in blue. (**b**) CD spectra of purified CL2-Af_EGFR_ or CL2-scFv(PD-L1) in 10 mM potassium phosphate buffer (pH 7.2).

**Figure S6. WB analysis of IGF2R, EGFR, Sortilin, and TfR expression in HeLa and MDA-MB-231 cells.** GAPDH served as a loading control. Blots are representative of three independent experiments.

**Figure S7. Verification of one bioorthogonal protein pair self-assembly via nickel bead pull-down assay.** (**a)** Schematic illustration of the Ni-NTA pull-down assay used to assess formation and stability of the IGF2-Im2/CL2-Af_EGFR_ complex. (**b)** SDS-PAGE analysis of pull-down fractions. FL, flow-through; E1, elution with 10 mM imidazole; E2, elution with 200 mM imidazole; E3, elution with 500 mM imidazole. The final two lanes show individual protein controls: untagged IGF2-Im2 and His-tagged CL2-Af_EGFR_.

**Figure S8. Dose-dependent degradation of EGFR by IGF2-Im2/CL2-Af_EGFR_ and IGF2-Af_EGFR_ in HeLa cells. (a)** WB analysis of EGFR degradation in HeLa cells treated with IGF2-Im2/CL2-Af_EGFR_ or IGF2-Af_EGFR_ at indicated concentrations (0-1000 nM) for 48 h. (**b)** Dose-dependent EGFR degradation in HeLa cells treated with IGF2-Im2/CL2-Af_EGFR_ at indicated concentrations (0–1000 nM) for 72 h. GAPDH served as a loading control. Blots are representative of three independent experiments.

**Figure S9**. IGF2R knockout abolishes EGFR degradation by the Type‑I UPTAB degrader. (**a**) Western blot analysis of IGF2R levels in HeLa cells after treatment with three independent siRNAs targeting IGF2R; NC (negative control) served as a non‑targeting control RNA. b-c) Western blot analysis of EGFR degradation in HeLa wild‑type (WT) cells (**b**) and HeLa IGF2R knockout (KO) cells (**c**) after treatment with increasing concentrations of IGF2‑Im2/CL2‑Af_EGFR_ (0–250 nM, 24 h). GAPDH served as a loading control. Blots are representative of three independent experiments.

**Figure S10. WB analysis of IGF2R and EGFR degradation in HeLa (a) and 5637 (b) cells treated with IGF2-Im2/CL2-Af_EGFR_ at indicated concentrations (0-1000 nM) for 48 h.** GAPDH served as a loading control. Blots are representative of three independent experiments.

**Figure S11**. Western blots of EGFR, pEGFR and pAkt in HeLa cells following incubation with CL2-Af_EGFR_ (100-500 nM) or IGF2-Im2/CL2-Af_EGFR_ for 48 h and 1 h of stimulation with 50 ng/ml of EGF. GAPDH served as a loading control. Blots are representative of three independent experiments.

**Figure S12. WB analysis of EGFR degradation by the inhibitor, degrader, and DDC in MDA-MB-231 cells.** (**a)** Concentration-dependent degradation of EGFR in MDA-MB-231 cells treated with IGF2-Im2^MMAE^/CL2-Af_EGFR_ (DDC) at indicated concentrations (0-1000 nM) for 48 h. Cells were incubated with the pre-assembled complex at a 1:1 molar ratio. (**b)** Comparison of EGFR degradation in MDA-MB-231 cells treated with CL2-Af_EGFR_ alone (inhibitor), IGF2-Im2/CL2-Af_EGFR_ (degrader), or IGF2-Im2^MMAE^/CL2-Af_EGFR_ (DDC) at 1000 nM for 48 h. GAPDH served as a loading control. Blots are representative of three independent experiments.

**Figure S13.** Live/dead staining and fluorescence imaging in 786-O cells. Cells were treated with PBS, CL2-Af_EGFR_ (500 nM), IGF2-Im2/CL2-Af_EGFR_ (500 nM), and IGF2-Im2^MMAE^/CL2-Af_EGFR_ (500 nM) for 48 h before analysis. Live cells were stained with Calcein-AM (green fluorescence, upper panels), and dead cells were stained with propidium iodide (red fluorescence, lower panels). Scale bar = 20 *μ*m.

**Figure S14.** Cytotoxicity of EGFR‑targeted constructs in non‑malignant HEK293 cells and comparison with cancer cell lines. (**a**) Cytotoxicity of CL2-Af_EGFR_, IGF2-Im2/CL2-Af_EGFR_, IGF2-Im2^MMAE^ and IGF2-Im2^MMAE^/CL2-Af_EGFR_ in non-malignant cell lines (HEK293) after 72 h treatment (n = 3 independent experiments). (**b**) Direct comparison of cytotoxicity induced by IGF2-Im2^MMAE^/CL2-Af_EGFR_ between cancer cell lines (HeLa, 786-O, and 5637) and non‑malignant (HEK293) cells (n = 3 independent experiments). Cell viability was assessed by standard assays and expressed as percentage relative to untreated controls. Data are shown as mean ± SD.

**Figure S15**. **Western blot analysis of several membrane proteins in HEK293 (a) and MDA-MB-231 cells (b) after treatment with CL2-Af_EGFR_, IGF2-Im2/CL2-Af_EGFR_, and IGF2-Im2^MMAE^/CL2-Af_EGFR_ (1000 nM) for 48 h. GAPDH served as a loading control. The Western blot plots are representative of three independently replicated samples.**

**Figure S16.** Structure of IGF2-Im2-Im7 predicted by AlphaFold3. The red domain represents IGF2, while the yellow domain represents Im2, and green domain represents Im7, GS linkers are labelled in blue.

**Figure S17. Identification of protein purity and complex formation of Type-II UPTABs.** (**a)** SDS-PAGE analysis confirming the purity and identity of recombinant proteins used for Type-II UPTAB assembly: IGF2-Im2-Im7, CL2-Af_EGFR_, and CL7-Nb*_c_*_-MET_. All proteins migrated at positions consistent with their predicted molecular weights. **(b)** SEC analysis of complex formation following incubation of IGF2-Im2-Im7 with CL2-Af_EGFR_ and CL7-Nb_c-MET_. Absorbance was monitored at 280 nm. The elution profile reveals a single, well-defined peak corresponding to the assembled ternary complex.

**Figure S18.** Structural prediction and CD spectroscopy analysis of CL7-Nb_c-MET_ protein. (**a**) Structure of CL7-Nb_c-MET_ predicted by AlphaFold3. The pink domain represents CL7, while the green domain represents Nb_c-MET_. The GS linker is labelled in blue. (**b**) The CD spectrum of CL7-Nb_c-MET_ in 10 mM potassium phosphate buffer (pH 7.2).

**Figure S19. Verification of two bioorthogonal protein pairs self-assembly via nickel bead pull-down assay.** (**a)** Schematic illustration of the Ni-NTA pull-down assay used to assess formation and stability of the IGF2-Im2-Im7/CL2-Af_EGFR_/CL7-Nb_c-MET_ complex. (**b)** SDS-PAGE analysis of pull-down fractions. FL, flow-through; E1, elution with 10 mM imidazole; E2, elution with 200 mM imidazole; E3, elution with 500 mM imidazole. The final three lanes show individual protein controls: untagged IGF2-Im2-Im7, untagged CL7-Nb_c-MET_ and His-tagged CL2-Af_EGFR_.

**Figure S20. The investigation of targeted protein degradation across IGF2R-deficient cell lines. (a) Scanning of IGF2R level in MDA-MB-231 cells after siRNA transfection. (b) Western blot analysis of EGFR and c-MET levels in MDA-MB-231 IGF2R-KO cells after treatment with IGF2-Im2-Im7/CL2-Af_EGFR_/CL7-Nb_c-MET_ (0-1000 nM, 24 h). c) Scanning of IGF2R level in A549 and HEK293 cells. HEK293 was shown as a control for comparison. d) Western blot analysis of EGFR and c-MET levels in A549 cells after treatment with IGF2-Im2-Im7/CL2-Af_EGFR_/CL7-Nb_c-MET_ (0-1000 nM).** GAPDH served as a loading control. Blots are representative of three independent experiments.

**Figure S21. Identification of protein purity and complex formation of Type-III UPTABs.** (**a)** SDS-PAGE analysis confirming the purity and identity of recombinant proteins used for Type-III UPTAB assembly: IGF2-Im2-Im7-Im9, CL2-Af_EGFR_, CL7-Nb_c-MET_ and CL9-Nb_HER2_. All proteins migrated at positions consistent with their predicted molecular weights. (**b)** SEC analysis of complex formation following incubation of IGF2-Im2-Im7-Im9, CL2-Af_EGFR_, CL7-Nb_c-MET_ and CL9-Nb_HER2_. Absorbance was monitored at 280 nm. The elution profile reveals a single, well-defined peak corresponding to the assembled quaternary complex.

**Figure S22.** Structural prediction and CD spectroscopy analysis of the CL9-Nb_HER2_ protein. (**a**) Structure of CL9-Nb_HER2_ predicted by AlphaFold3. The pink domains represent CL9, while the green domains represent Nb_HER2_. The GS linker is labelled in blue. **(b)** The CD spectrum of CL9-Nb_HER2_ in 10 mM potassium phosphate buffer (pH 7.2).

**Figure S23. Verification of three bioorthogonal protein pairs self-assembly via nickel bead pull-down assay.** (**a)** Schematic illustration of the Ni-NTA pull-down assay used to assess formation and stability of the IGF2-Im2-Im7-Im9/CL2-Af_EGFR_/CL7-Nb_c-MET_ /CL9-Nb_HER2_ complex. (**b)** SDS-PAGE analysis of pull-down fractions. FL, flow-through; E1, elution with 10 mM imidazole; E2, elution with 200 mM imidazole; E3, elution with 500 mM imidazole. The final four lanes show individual protein controls: untagged IGF2-Im2-Im7, untagged CL7-Nb_c-MET_, untagged CL9-Nb_HER2_ and His-tagged CL2-Af_EGFR_.

**Figure S24. The investigation of targeted protein degradation across IGF2R-deficient cell lines. (a) Western blot analysis of HER2, EGFR and c-MET levels in MDA-MB-231 IGF2R-KO cells after treatment with IGF2-Im2-Im7-Im9/CL2-Af_EGFR_/CL7-Nb_c-MET_/CL9-Nb_HER2_ (0-1000 nM, 24 h). (b) Western blot analysis of HER2, EGFR and c-MET levels in A549 cells after treatment with IGF2-Im2-Im7-Im9/CL2-Af_EGFR_/CL7-Nb_c-MET_/CL9-Nb_HER2_ (0-1000 nM).** GAPDH served as a loading control. Blots are representative of three independent experiments.

**Figure S25. Anti-proliferative effect of Type-III DDC on HepG2 and 5637 cancer cell lines.** Cells were treated with indicated formulations for 72 h, and cell viability was assessed by CCK-8 assay. IGF2-279 denotes IGF2-Im2-Im7-Im9; CL2 denotes CL2-Af_EGFR_; CL7 denotes CL7-Nb_c-MET_; CL9 denotes CL9-Nb_HER2_. Data are presented as mean ± SD (n = 3 independent experiments).

**Section 4 Amino acid sequence of proteins**

**IGF2-S-Im2**

AYRPSETLCGGELVDTLQFVCGDRGFYFSRPASRVSRRSRGIVEECCFRSCDLALLETYCATPAKSEGGGGSELKHSISDYTEAEFLEFVKKICRAEGATEEDDNKLVREFERLTEHPDGSDLIYYPRDDREDSPEGIVKEIKEWRAANGKSGFKQGLE

**IGF2-Im2**

AYRPSETLCGGELVDTLQFVCGDRGFYFSRPASRVSRRSRGIVEECCFRSCDLALLETYCATPAKSEGGGGSGGGGSELKHSISDYTEAEFLEFVKKICRAEGATEEDDNKLVREFERLTEHPDGSDLIYYPRDDREDSPEGIVKEIKEWRAANGKSGFKQGLE

**IGF2-L-Im2**

AYRPSETLCGGELVDTLQFVCGDRGFYFSRPASRVSRRSRGIVEECCFRSCDLALLETYCATPAKSEGGGGSGGGGSGGGGSELKHSISDYTEAEFLEFVKKICRAEGATEEDDNKLVREFERLTEHPDGSDLIYYPRDDREDSPEGIVKEIKEWRAANGKSGFKQGLE

**Tf-Im2**

KKFIKELLEQGYSKEETAIKVVQKFNVSVVVVTDDEEKAKEIAEYIKKNVPSATVVVYENLIVAKVESHEESTKVWELAQKAYGGGGSGGGGSELKHSISDYTEAEFLEFVKKICRAEGATEEDDNKLVREFERLTEHPDGSDLIYYPRDDREDSPEGIVKEIKEWRAANGKSGFKQGLE

**Nb_Sortilin_-Im2**

SFLLKHAVRVIERLLEEGNVELAEKWVEQLKRLAHAQRDPEALRRAEELEEIVEELLEGGGGSGGGGSELKHSISDYTEAEFLEFVKKICRAEGATEEDDNKLVREFERLTEHPDGSDLIYYPRDDREDSPEGIVKEIKEWRAANGKSGFKQGLE

**IGF2-Im2-Im7**

AYRPSETLCGGELVDTLQFVCGDRGFYFSRPASRVSRRSRGIVEECCFRSCDLALLETYCATPAKSEGGGGSGGGGSELKHSISDYTEAEFLEFVKKICRAEGATEEDDNKLVREFERLTEHPDGSDLIYYPRDDREDSPEGIVKEIKEWRAANGKSGFKQGLEGGGGSGGGGSELKNSISDYTEAEFVQLLKEIEKENVAATDDVLDVLLEHFVKITEHPDGTDLIYYPSDNRDDSPEGIVKEIKEWRAANGKPGFKQG

**IGF2-Im2-Im7-Im9**

AYRPSETLCGGELVDTLQFVCGDRGFYFSRPASRVSRRSRGIVEECCFRSCDLALLETYCATPAKSEGGGGSGGGGSELKHSISDYTEAEFLEFVKKICRAEGATEEDDNKLVREFERLTEHPDGSDLIYYPRDDREDSPEGIVKEIKEWRAANGKSGFKQGLEGGGGSGGGGSELKNSISDYTEAEFVQLLKEIEKENVAATDDVLDVLLEHFVKITEHPDGTDLIYYPSDNRDDSPEGIVKEIKEWRAANGKPGFKQGGGGGSGGGGSMELKHSISDYTEAEFLQLVTTICNADTSSEEELVKLVTHFEEMTEHPSGSDLIYYPKEGDDDSPSGIVNTVKQWRAANGKSGFKQG

**CL2-sfGFP**

MSKSNEPGKATGEGKPVGDKWLDDAGKDSGAPIPDRIADKLRDKEFENFDDFRETFWEEVSKDPDLSKQFKGSNKTNIQKGKAPFARKKDQVGGRERFELNHQKPIEQDGGVYDMNNIRVTTPKRNIDIEGGGGGSGGGGSMVSKGEELFTGVVPILVELDGDVNGHKFSVRGEGEGDATNGKLTLKFICTTGKLPVPWPTLVTTLTYGVQCFSRYPDHMKQHDFFKSAMPEGYVQERTISFKDDGTYKTRAEVKFEGDTLVNRIELKGIDFKEDGNILGHKLEYNFNSHNVYITADKQKNGIKAEFEIRHNVEDGSVQLADHYQQNTPIGDGPVLLPDDHYLSTESVLSKDPNEDRDHMVLLEFVTAAGIDLGMDELYKHHHHHHS

**CL2-S-Af_EGFR_**

MSKSNEPGKATGEGKPVGDKWLDDAGKDSGAPIPDRIADKLRDKEFENFDDFRETFWEEVSKDPDLSKQFKGSNKTNIQKGKAPFARKKDQVGGRERFELNHQKPIEQDGGVYDMNNIRVTTPKRNIDIEGGGGGSVDNKFNKEMWAAWEEIRNLPNLNGWQMTAFIASLVDDPSQSANLLAEAKKLNDAQAPKHHHHHHS

**CL2-Af_EGFR_**

MSKSNEPGKATGEGKPVGDKWLDDAGKDSGAPIPDRIADKLRDKEFENFDDFRETFWEEVSKDPDLSKQFKGSNKTNIQKGKAPFARKKDQVGGRERFELNHQKPIEQDGGVYDMNNIRVTTPKRNIDIEGGGGGSGGGGSVDNKFNKEMWAAWEEIRNLPNLNGWQMTAFIASLVDDPSQSANLLAEAKKLNDAQAPKHHHHHHS

**CL2-L-Af_EGFR_**

MSKSNEPGKATGEGKPVGDKWLDDAGKDSGAPIPDRIADKLRDKEFENFDDFRETFWEEVSKDPDLSKQFKGSNKTNIQKGKAPFARKKDQVGGRERFELNHQKPIEQDGGVYDMNNIRVTTPKRNIDIEGGGGGSGGGGSGGGGSVDNKFNKEMWAAWEEIRNLPNLNGWQMTAFIASLVDDPSQSANLLAEAKKLNDAQAPKHHHHHHS

**CL2-scFv(PDL1)**

MSKSNEPGKATGEGKPVGDKWLDDAGKDSGAPIPDRIADKLRDKEFENFDDFRETFWEEVSKDPDLSKQFKGSNKTNIQKGKAPFARKKDQVGGRERFELNHQKPIEQDGGVYDMNNIRVTTPKRNIDIEGGGGGSGGGGSQSALTQPASVSGSPGQSITISCTGTSSDVGGYNYVSWYQQHPGKAPKLMIYDVSNRPSGVSNRFSGSKSGNTASLTISGLQAEDEADYYCSSYTSSSTRVFGTGTKVTVLGGGGSGGGGSGGGGSGGGGSEVQLLESGGGLVQPGGSLRLSCAASGFTFSSYIMMWVRQAPGKGLEWVSSIYPSGGITFYADTVKGRFTISRDNSKNTLYLQMNSLRAEDTAVYYCARIKLGTVTTVDYWGQGTLVTVSSHHHHHHS

**CL7-Nb_c-MET_**

MSKSNEPGKATGEGKPVNNKWLNNAGKDLGSPVPDRIANKLRDKEFESFDDFRETFWEEVSKDPELSKQFSRNNNDRMKVGKAPKTRTQDVSGKRTSFELNHQKPIEQNGGVYDMDNISVVTPKRNIDIEGGGGGSGGGGSQVQLQESGGGSVQAGGSLKLTCAASGYIFNSCGMGWYRQSPGRERELVSRISGDGDTWHKESVKGRFTISQDNVKKTLYLQMNSLKPEDTAVYFCAVCYNLETYWGQGTQVTVSS

**CL9-Nb_HER2_**

MSKSNEPGKATGEGKPVGDKWLDDAGKDSGAPIPDRIADKLRDKEFESFDDFRETVWEEVSKDPELSKNLNPSNKSSVSKGYSPFTPKNQQVGGRKVYELNHQKPIEQGGEVYDMDNIRVTTPKRNIDIEGGGGSGGGGSEVQLVESGGGLVQPGGSLRLSCEASGFNFERYDMSWVRQAPGKGPEWVSRLNSFGRSTYYLDSVKGRFTVSRDNAQNMLYLQMNNLKPEDTAVYYCARSTDVSPGLSSWWTYEYDVWGQGTQVTVSS

IGF2-Af_EGFR_

AYRPSETLCGGELVDTLQFVCGDRGFYFSRPASRVSRRSRGIVEECCFRSCDLALLETYCATPAKSEGGGGSGGGGSVDNKFNKEMWAAWEEIRNLPNLNGWQMTAFIASLVDDPSQSANLLAEAKKLNDAQAPK

**Section 5 Appended WB images**
